# Recovery of Lithium Carbonate from Dilute Li-Rich Brine via Homogenous and Heterogeneous Precipitation

**DOI:** 10.1021/acs.iecr.2c01397

**Published:** 2022-08-30

**Authors:** Giuseppe Battaglia, Leon Berkemeyer, Andrea Cipollina, José Luis Cortina, Marc Fernandez de Labastida, Julio Lopez Rodriguez, Daniel Winter

**Affiliations:** † Dipartimento di Ingegneria, Università degli Studi di Palermo (UNIPA), viale delle Scienze Ed.6, Palermo 90128, Italy; ‡ Fraunhofer Institute for Solar Energy Systems ISE, Heidenhofstraße 2, Freiburg 79110, Germany; § Chemical Engineering Department, Escola d′Enginyeria de Barcelona Est (EEBE), Universitat Politècnica de Catalunya (UPC)-BarcelonaTECH, C/Eduard Maristany 10−14, Campus Diagonal-Besòs, Barcelona 08930, Spain

## Abstract

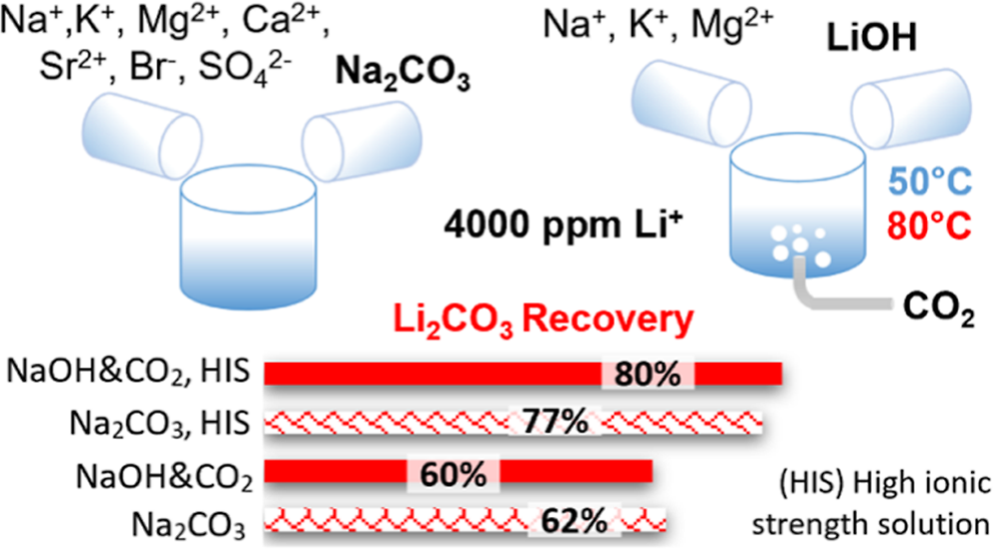

An extensive experimental campaign on Li recovery from relatively dilute LiCl solutions (i.e., Li^+^ ∼ 4000 ppm) is presented to identify the best operating conditions for a Li_2_CO_3_ crystallization unit. Lithium is currently mainly produced via solar evaporation, purification, and precipitation from highly concentrated Li brines located in a few world areas. The process requires large surfaces and long times (18–24 months) to concentrate Li^+^ up to 20,000 ppm. The present work investigates two separation routes to extract Li^+^ from synthetic solutions, mimicking those obtained from low-content Li^+^ sources through selective Li^+^ separation and further concentration steps: (i) addition of Na_2_CO_3_ solution and (ii) addition of NaOH solution + CO_2_ insufflation. A Li recovery up to 80% and purities up to 99% at 80 °C and with high-ionic strength solutions was achieved employing NaOH solution + CO_2_ insufflation and an ethanol washing step.

## 1 Introduction

The increasing demand of raw materials has pushed researchers and industrials to seek for new alternative solutions to overcome the limited availability from typical sources (e.g., mines and ores). Seawater, brines, and bitterns have been extensively studied as promising alternatives for the extraction and recovery of valuable and crucial elements^1−4^ such as magnesium (Mg^2+^), lithium (Li^+^), rubidium (Rb^+^), strontium (Sr^+^), and so forth. Seawater contains almost all the elements of the periodic table, although many elements are present in very low concentrations. Seawater bitterns, such as those generated in saltworks, are more concentrated than seawater. Within saltworks, seawater goes through a natural process of evaporation and fractional crystallization, aiming at producing sea salt and very concentrated brine (bittern) as a byproduct.^5^

Lithium, recently defined as “the new white gold”,^6^ is extensively employed for the production of lithium-ion batteries, which are widely used thanks to their high specific energy density (100–265 W h/kg) and lifespan cycles (400–1200), making them the most suitable technology for electrical vehicles and portable electronic devices.^7^ The industrial lithium demand has increased sharply, and it is foreseen to increase from 237,000 tons of lithium carbonate equivalent (LCE) in 2018 to 4.4–7.5 million tons of LCE by 2100.^8^ Li^+^ is the 14th most abundant element in seawater with an average concentration of 0.17 ppm. From statistics, it can be estimated that a total amount of elementary lithium between 230,000 and 250,000 megatons (Mt) is contained in seawater,^9^ equivalent to 1,200,000–1,300,000 Mt of lithium carbonate (LCE), thus orders of magnitude higher than present and future world demand. However, novel and innovative processes have to be developed to recover and extract Li^+^ from low-grade and unfavorable sources. So far, most of the exploited world’s Li^+^ reserves are high-content Li^+^ brines located at few geographically specific sites, for example, Chile, Bolivia, China, and Argentina.^6,8^ An example is the Salar de del Hombre Muerto brines (north-western Argentina) that contain more than 1000 ppm Li^+^.^10^

**Figure 1:**
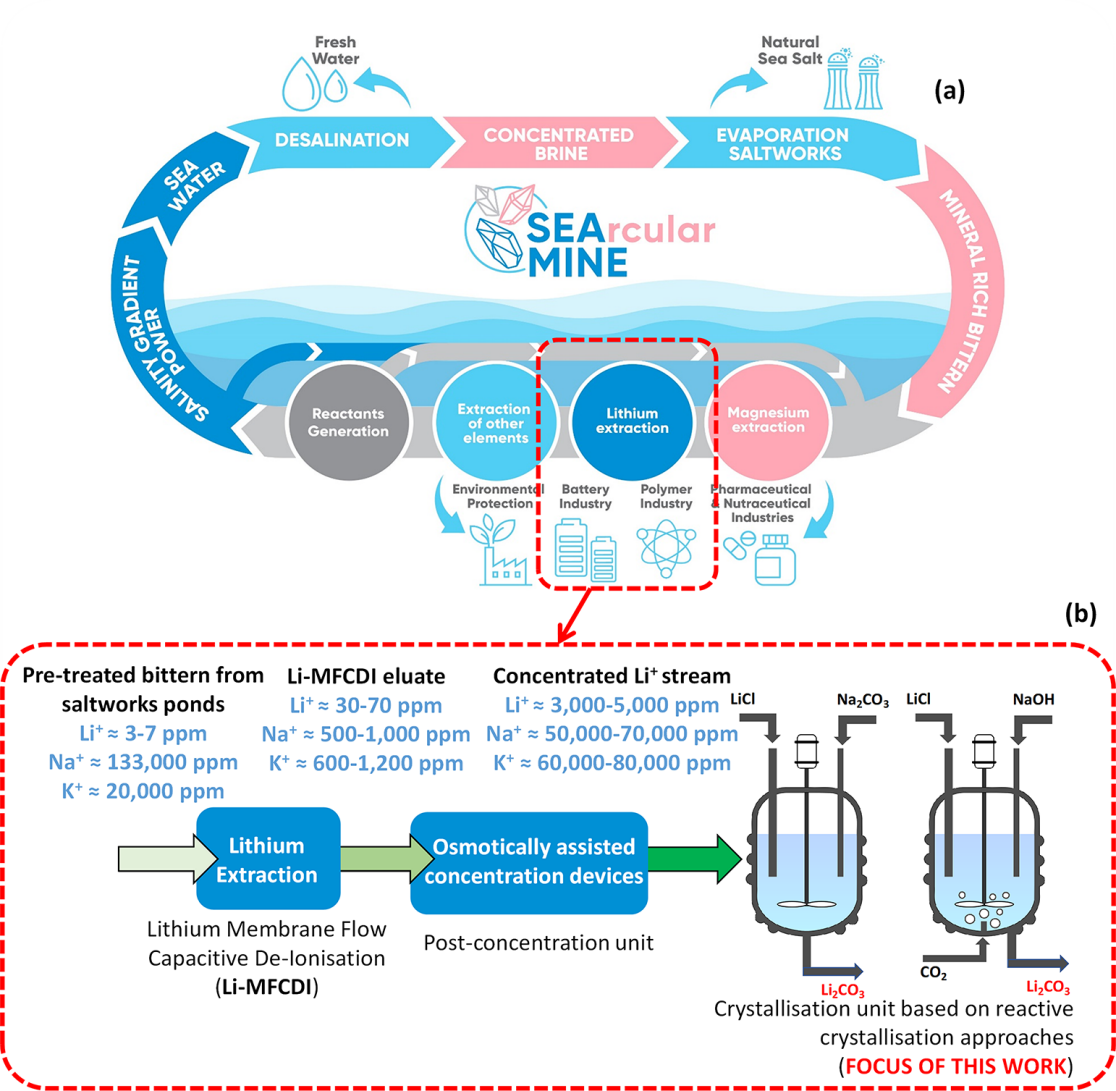
Schematic representation of the general SEArcularMINE-integrated treatment chain (a) and a detailed description of the lithium separation/concentration/recovery steps within the chain (b).

In the last 20 years, research efforts have been put for the development of novel processes for the recovery of lithium from low-grade and unfavorable deposits as for lithium end-life waste batteries,^11−14^ wastewaters from oil and gas fields,^15^ and low-lithium-content brines/bitterns.^16−18^ Although Li^+^ content in bitterns is lower than that in salty brines reserves, as it reaches values from 2–3 ppm up to 20 ppm in Egyptian bitterns,^16^ saltwork bitterns are generated every year starting from seawater and are, therefore, a more sustainable and continuous source of Li^+^ compared to salty brines accumulated in thousands of years. In this context, the SEArcularMINE European project aims at valorizing spent bitterns produced by the traditional and still widely employed saltworks (a schematic of the SEArcularMINE-integrated treatment chain is shown in Figure 1a. Among the other minerals, lithium is going to be recovered for the first time employing a novel membrane-based electrochemical Li^+^ separator (Li-MFCDI), which separates lithium ions from the bittern into a receiving solution. The Li-rich MFCDI eluate is further concentrated using osmotically assisted concentration devices, and finally, the Li^+^-concentrated solution is fed into a crystallizer unit to recover Li^+^ in the form of carbonate salt (a scheme of the lithium separation/concentration/recovery steps within the chain is shown in Figure 1b). The overall Li^+^ recovery stage allows concentrating the Li^+^ from 3 to 7 ppm, in the original bittern, to a final concentration of 3000–5000 ppm, thus enabling the possibility of solids separation in the crystallizer. It is worth noting that the Li-MFCDI separator is not expected to be ideally selective toward the passage of Li^+^, especially with the extremely high starting concentration of other monovalent ions; thus, a significant presence of other ions in the Li-MFCDI eluate is expected too, within the range of concentration qualitatively indicated in the scheme in Figure 1b.

### 1.1 Overview of Current Strategies for Li_2_CO_3_(s) Production and Motivation of This Work

The most important commercial Li^+^ compound is Li_2_CO_3_(s) that accounts for 60% of the market share of lithium-based commercial products,^19^ followed by lithium hydroxide LiOH(s) (23% market share).^7^

Starting from Li-rich brines, the major process for recovering lithium from brines is the lime soda evaporation process that typically consists of stages starting with concentration by evaporation, impurity removal, and precipitation. Li^+^ is then recovered by using soda ash (Na_2_CO_3_) to obtain Li_2_CO_3_ with a 99.5% purity. In Section 3.4, several precipitation approaches using Na_2_CO_3_ as a precipitant agent are discussed. Further processes based on adsorption, precipitation, and on ion exchange/solvent extraction processes were also presented in the literature.^16,20,21^

The possibility of using CO_2_ to recover lithium as a contribution to the circular economy and environmental sustainability was also addressed in the literature by several fundamental studies, which, however, have not been brought to the testing level by the proposed precipitation route with real Li-rich brines. Matsumoto^22^ used a waveguide-type microwave apparatus to produce CO_2_ microbubbles in an aqueous solution containing lithium ions (starting from LiNO_3_ salt) to obtain lithium carbonate (Li_2_CO_3_(s)) nanoparticles. Sun et al.^23^ employed a spinning disk reactor to precipitate Li_2_CO_3_(s) by gas–liquid reactive crystallization of LiOH and CO_2_ using an ultrasound field. The ultrasound field, the temperature, and the CO_2_ flow rate were found to significantly influence the Li_2_CO_3_(s) particle size. The use of a falling film column was also investigated, some years later, by Sun et al.^24^ for the same Li_2_CO_3_(s) precipitation process in the LiOH–CO_2_ system. Tian et al.^25^ studied the influence of ammonium hydroxide (NH_3_·H_2_O) in the gas–liquid reactive crystallization of Li_2_CO_3_(s). The ammonium ions were believed to greatly influence the Li_2_CO_3_(s) precipitation process by inhibiting the re-carbonation of Li_2_CO_3_(s). Zhou et al.^26^ used a coupled reaction and solvent extraction process to produce Li_2_CO_3_(s) from the LiCl and CO_2_(g) system. HCl was removed, to increase the reaction yield, by solvent extraction using tri-*n*-octyl amine and iso-octanol as solvent. Han et al.^19^ presented a comparison between homogenous Li_2_CO_3_ precipitation using only soda ash and heterogeneous Li_2_CO_3_ precipitation employing NaOH and the addition of CO_2_(g) from Li_2_SO_4_ solutions mimicking a waste solution of lithium-containing electrical and electronic equipment. Results showed that both methods can be feasible to recover lithium as lithium carbonate salt from Li_2_SO_4_ solutions.

On the basis of the above literature review, it is clear how the Li_2_CO_3_ precipitation process has been extensively studied in the past. However, Li^+^ precipitation has been mostly studied in highly Li-concentrated solutions, with Li^+^ concentrations higher than 10,000 ppm,^11,19,23,27^ with less studies addressing low Li-containing ones, with concentrations lower than 5000 ppm (as in ref (28)). Nevertheless, lithium extraction from seawater, brines, and bitterns requires a preliminary concentration step to increase lithium concentrations from tens to thousands of ppm, highlighting the importance of characterizing the precipitation phenomena at low concentration than in conventional processes.

The present paper aims at reporting an extensive experimental campaign to prove the feasibility and provide the most favorable strategies for the recovery of Li^+^ from low-concentration solutions (Li^+^ concentration ∼ 4000 ppm). Here, attention is on Li^+^ recovery and purity determined in several precipitation cases. Specifically, Li_2_CO_3_(s) precipitation was studied following two precipitation routes: (i) using Na_2_CO_3_ solution and NaOH solution and CO_2_(g) insufflation. Several parameters affecting both precipitation routes were investigated, such as Li^+^/precipitant ratios, solution temperature, and the presence of dissolved monovalent and divalent ions, which can be present in the eluate of Li-MFCDI from the feed bittern (e.g., Na^+^, K^+^, Cl^–^, SO_4_^2–^, etc.) and could be further concentrated before crystallization. A purification step using ethanol was also studied to enhance Li_2_CO_3_ solid purity.

In regard to the NaOH solution and CO_2_(g) insufflation route, to the best of the author knowledge’s, there are no other studies reporting Li^+^ purity and recovery in Li solutions containing dissolved monovalent and divalent ions mimicking real Li^+^ solutions. Results provide straightforward and useful information for the design of Li_2_CO_3_ crystallizers for the recovery of lithium from low-Li-concentration solutions.

## 2 Materials and Methods

All precipitation experiments were performed on a laboratory-scale setup, preparing synthetic solutions of LiCl, plus other salts (as simulated feed brine) and Na_2_CO_3_ or NaOH as precipitation inducing reactants. Details on materials, experimental setups, and procedures are reported in the following sections, while for the sake of brevity, a complete description of the two investigated precipitation routes and a literature overview of previous studies focused on Li_2_CO_3_ precipitation fundamentals are reported in the Supporting Information.

### 2.1 Materials

Table S1 in the Supporting Information lists all chemicals used in the Li^+^ precipitation experiments. The reagents were of analytical grade and were employed without further purification. Deionized water was used for all experiments. Synthetic solutions were prepared by dissolving the desired salts weighted using a precision balance (Sartorius BCE 653) in a beaker filled with deionized water to a defined total mass of salts and water of ∼110 g. The precise mass for each experiment is reported in the relevant tables in the Results and Discussion section. The total volume was determined by measuring the solution density with a DMA 35 density meter (Anton Paar) and knowing the total mass of the solution. LiCl solutions of ∼5000 ppm (0.70 M) were prepared aiming at obtaining an initial Li^+^ concentration of ∼4000 ppm (0.59 M) after reactant solution addition (which generates a further dilution of the initial feed solution at time *t*_o_, at which reaction has not started yet due to the low precipitation kinetics). Exact concentrations for each experiment are reported in the relevant tables in the Results and Discussion section.

### 2.2 Experimental Setup and Procedure for Li^+^ Precipitation with Na_2_CO_3_

**Figure 2:**
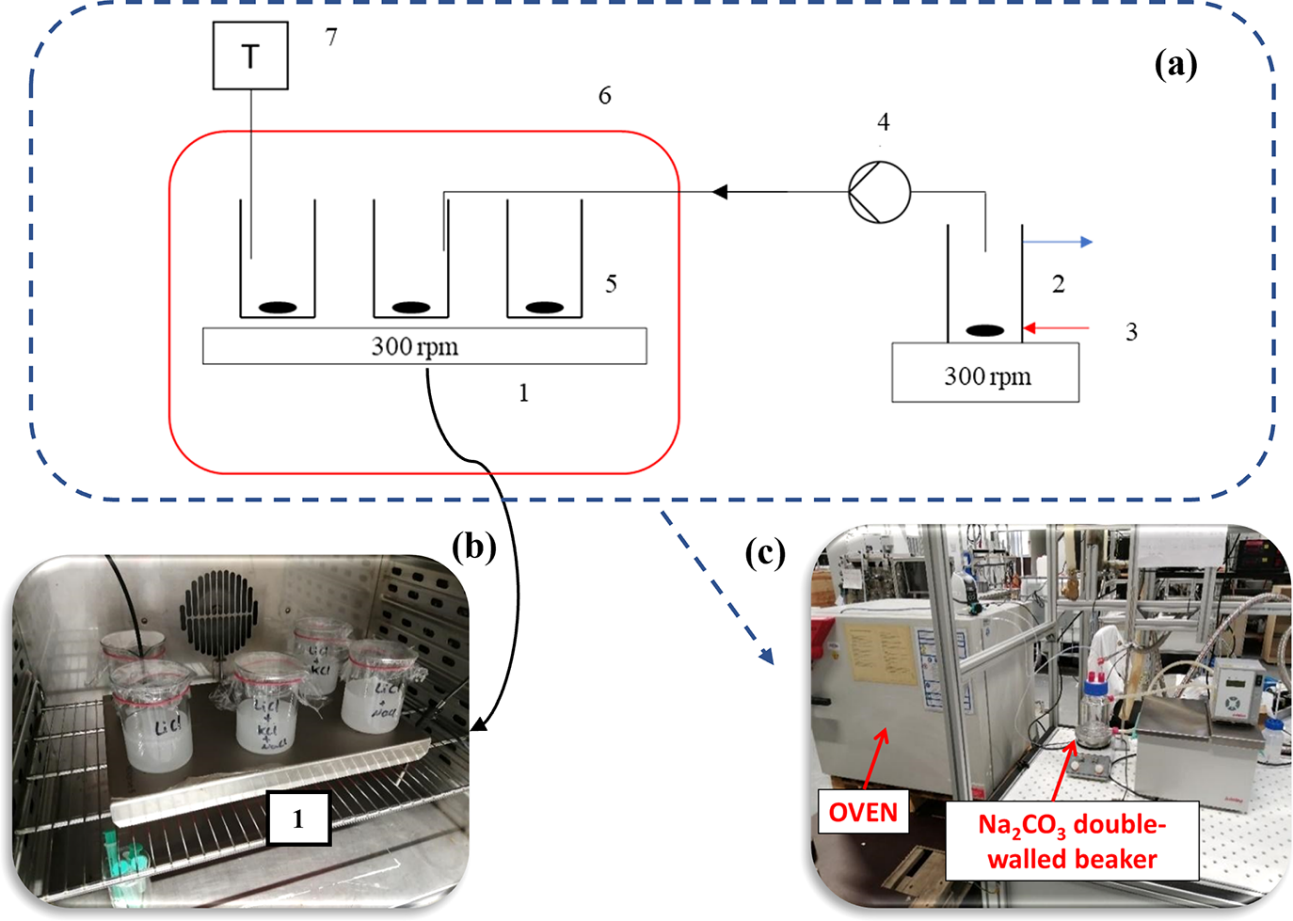
(a) Schematic representation of the employed experimental setup for lithium precipitation with sodium carbonate: (1) six-position magnetic stirrer, (2) double-walled beaker, (3) heating water from a thermostatic bath, (4) peristaltic pump, (5) 250 mL volume beakers, (6) oven, (7) PT100 temperature probe. Pictures of the experimental setup; (b) six-position magnetic stirrer with precipitated lithium carbonate placed in an oven. (c) Whole experimental set up.

The employed experimental setup for Li_2_CO_3_ precipitation tests using Na_2_CO_3_ solutions is presented in Figure 2. The synthetic brines were stirred steadily in a thermostatic room on a six-position magnetic stirrer and covered with Parafilm to avoid evaporation losses. The temperature of the samples was indirectly checked by measuring the temperature of a blank sample consisting of a beaker filled with a comparable amount of water, via a Pt100 temperature probe. All solutions were stirred at a speed of 300 rpm. The temperature of the Na_2_CO_3_ solution, to be injected into the abovementioned samples, was controlled using a double-walled beaker connected to a thermostat and set to the same temperature as that of the thermostatic room where the precipitation took place. After reaching the desired constant temperature, the desired volume of a 2.0 M Na_2_CO_3_ solution was added to the Li^+^-containing solution with a peristaltic pump (SIMDOS 02) at a flow rate of 10 mL/min; the same flow rate and solution concentration were used in all the experiments, unless stated otherwise. In all experiments, the reaction time is considered to start after the complete addition of the Na_2_CO_3_ solution volume.

### 2.3 Experimental Setup and Procedure for Li^+^ Precipitation with NaOH and CO_2_(g)

**Figure 3:**
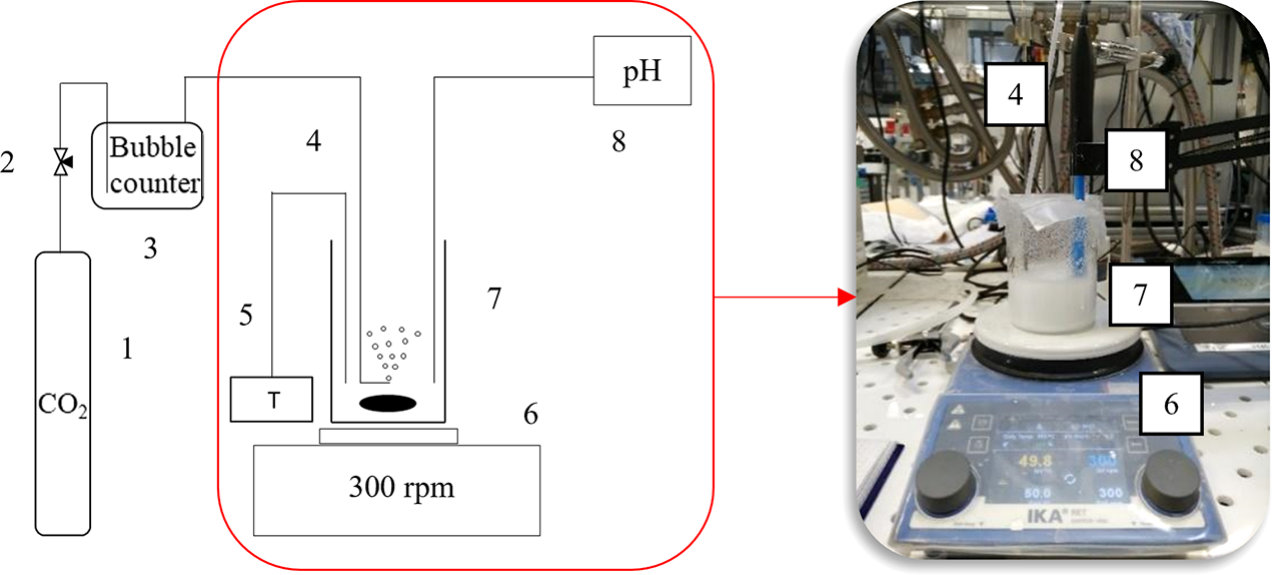
(a) Schematic representation of the experimental setup employed for lithium carbonate precipitation with sodium hydroxide and carbon dioxide insufflation: (1) carbon dioxide bottle, (2) needle valve, (3) bubble counter with a regulator, (4) PE hose for CO_2_ insufflation *Ø* 0.5 mm, (5) PT100 thermocouple probe (6) magnetic stirrer with a heating plate, (7) 250 mL beaker, (8) pH electrode with a measuring device. (b) Picture of the experimental setup during Li_2_CO_3_ precipitation.

The experimental setup employed for Li_2_CO_3_(s) precipitation with NaOH and CO_2_(g) insufflation is shown in Figure 3. In this case, an 8.0 M NaOH solution (32 % wt) was employed. The NaOH/LiCl solution was placed in a 250 mL beaker heated and stirred using a RET control-visc white stirrer from IKA, which offers a heating plate whose temperature is controlled based on a feedback signal acquired by a submersed Pt100 temperature probe. When the solution reached the desired temperature, CO_2_(g) was supplied through a polyethylene (PE) hose with an inner diameter of 0.5 mm. The hose was placed close to the stirrer to better disperse the gas bubbles and prevent any clogging. To minimize water losses due to evaporation, the beaker was covered with Parafilm. The CO_2_(g) feed rate was adjusted by using a needle valve and a downstream bubble counter. The pH was continuously monitored in the precipitation beaker via a temperature-compensated SenTix precision electrode from WTW.

### 2.4 Sampling and Analytical Procedures

For the quantitative determination of cation concentration in the reacting solution, from which Li^+^ recovery can be calculated, samples were withdrawn with pre-heated syringes (kept at the reaction temperature, to prevent any Li_2_CO_3_(s) dissolution). After sampling, the solution was filtered with a Berrytec nylon syringe filter (0.22 μm) and directly diluted 1:100 to interrupt the precipitation kinetics. The solutions were further diluted, and their composition was measured by employing a multiparameter optical emission spectrometer (ICP–OES, Varian 720-ES type).

Multiple determinations of individual measurement points were carried out with a standard deviation of 3%. ICP–OES measurement accuracy was also verified by comparing ICP–OES concentration, measured at the beginning of the experiment, with the one expected from the mass of lithium dissolved in the feed. A deviation lower or equal to 4% was determined in all cases. For the sake of graphical clarity in all plots, the relevant error bars are not reported as they would coincide with the size of the symbols.

To determine Li_2_CO_3_ solid purity, the precipitated solid samples were separated by vacuum filtration with a Büchner funnel using a cellulose acetate filter having a pore size of 0.45 μm. After filtration, the crystals were dried in a moisture analyzer (DLB-160-3A by Kern) at 105 °C for 12 h. Part of the dried precipitate was re-dissolved in a 1% HNO_3_ solution and further diluted with deionized water. Subsequently, the concentration of dissolved lithium was determined by ICP–OES (see above).

In selected experiments, the precipitate was washed in order to increase its purity. For this purpose, ∼0.1 g of Li_2_CO_3_ was weighted and then suspended in 50 mL of ethanol (*w* = 70%) solution at room temperature for 1 h. After this step, the precipitate was filtered again, and the purity in Li^+^ was determined by ICP–OES.

### 2.5 Precipitation Performance Parameters

In all the performed experiments, the recovery of lithium was assessed. It was calculated as the difference between the initial and final mass of lithium in solution divided by its initial mass (eq 1). The final solution volume was inferred as the sum of the volumes of the feed Li-rich brine and the precipitant solution (Na_2_CO_3_ or NaOH).
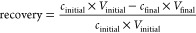
1

The mass purity of precipitate in Li^+^ was calculated according to eq 2
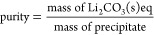
2where the equivalent mass of Li_2_CO_3_ was determined from the measured Li^+^ concentration in the collected precipitate samples (approximately 100 mg of the dried precipitate, see Section 2.4).

## 3 Results and Discussion

### 3.1 Lithium Precipitation with Na_2_CO_3_

The influence of several operating parameters on lithium precipitation using Na_2_CO_3_ was analyzed, addressing in particular (i) the effect of different CO_3_^2–^/Li^+^ molar ratios, (ii) the effect of solution temperature and ionic strength (given by NaCl and KCl dissolved salts) and (iii) the effect of the presence of divalent cations (namely, calcium, magnesium, and strontium) and anions (namely, sulfate and bromide ions) in the Li-rich feed brine.

#### 3.1.1 Influence of the [CO_3_^2–^]/[Li^+^] Ratio

The influence of the [CO_3_^2–^]/[Li^+^] operating ratio on Li^+^ recovery and purity was investigated. Five precipitation scenarios were carried out within the [CO_3_^2–^]/[Li^+^] range from 0.25 to 2 (mol/mol). Note that the [CO_3_^2–^]/[Li^+^] value of 0.5 represents the stoichiometric precipitation condition, while lower and upper ratio values refer to under- and over-stoichiometric conditions with respect to the excess or lack CO_3_^2–^ ions, respectively. A constant temperature of 50 °C and a 300 rpm stirring rate were maintained in all experiments. Details of the reacting quantities for each test are reported in Table S2 in the Supporting Information.

**Figure 4:**
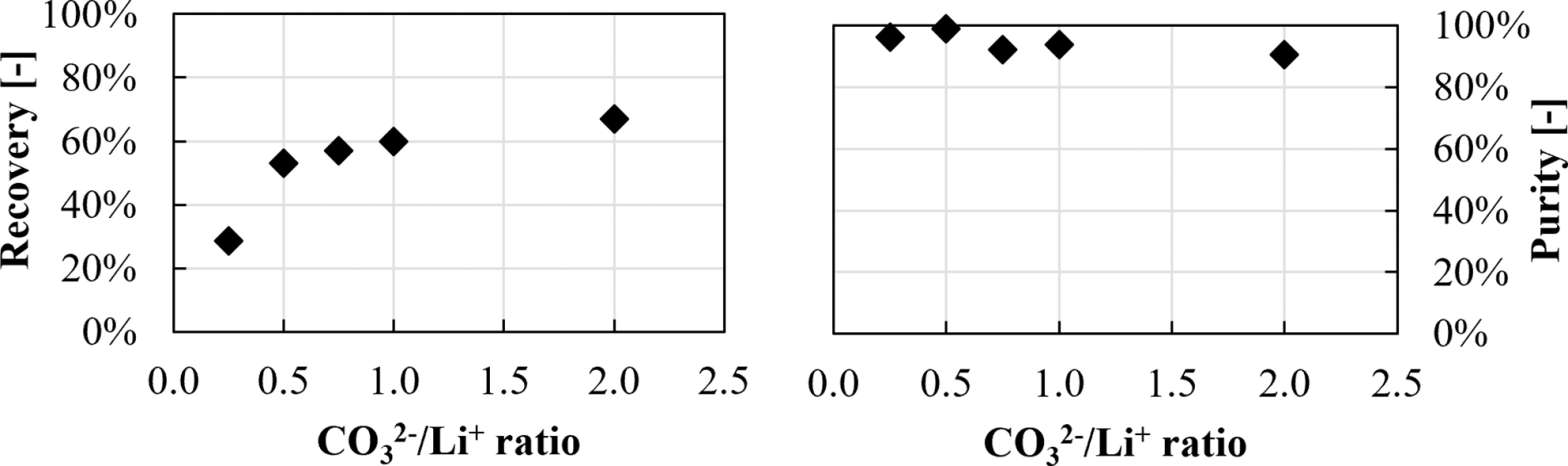
Li_2_CO_3_ recovery and purity as a function of the [CO_3_^2–^]/[Li^+^] ratio.

Li^+^ recovery, eq 1, and purity, eq 2, observed at the end of all experiments (after 2 h) are shown in Figure 4.

Li^+^ recovery significantly increases from ∼30 to ∼60% using a CO_3_^2–^/Li^+^ ratio of 0.25 and 1, respectively. On the other hand, only a slight increase is noticed when increasing the CO_3_^2–^/Li^+^ ratio from 1 to 2, that is, from ∼60 to ∼65%. Therefore, all the hereinafter reported experiments were carried out using a CO_3_^2–^/Li^+^ ratio of 1. Purity ranges between 98 and 90%, slightly decreasing at high CO_3_^2–^/Li^+^ ratios. In all these cases, the impurities are attributed mainly to trapped Na_2_CO_3_, remaining in the liquor entrained within the particle cakes after filtration.

#### 3.1.2 Influence of Temperature and Ionic Strength

Li_2_CO_3_(s) solubility decreases when the temperature is increased (see also the Supporting Information); thus, a beneficial effect of temperature on the precipitation rate is expected. In particular, the influence of temperature on the Li_2_CO_3_ precipitation process was studied by performing experiments at 50 °C and at 80 °C with and without the presence of other monovalent ions in solution, namely, Na^+^ and K^+^. The presence of dissolved ions (e.g., Na^+^ and K^+^) increases solution ionic strength, which can be calculated as
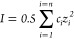
3where *I* is the solution ionic strength and *c*_*i*_ and *z*_*i*_ are the *i*-th ion concentration and valence, respectively.

Four precipitation tests were carried out using a starting (before Na_2_CO_3_ solution addition) 0.70 M LiCl solution (i) as a pure salt (*I* = 0.70 M) or with (ii) 1.5 M KCl (*I* = 2.20 M), (iii) 2.0 M NaCl (*I* = 2.70 M), and (iv) both 2.0 M NaCl and 1.5 M KCl (*I* = 4.20 M). Such NaCl and KCl concentrations were chosen based on preliminary calculation regarding the actual selectivity properties of the Li-MFCDI against monovalent and divalent ions present in the treated brine, as discussed in the introduction and shown in Figure 1. Details for all the four investigated cases are reported in Table S3 in the Supporting Information. In all experiments, solutions were stirred at 300 rpm and a double excess of a 2.0 M Na_2_CO_3_ solution (CO_3_^2–^/Li^+^ ratio of 1), fed at a flow rate of 10 mL/min, was employed.

**Figure 5:**
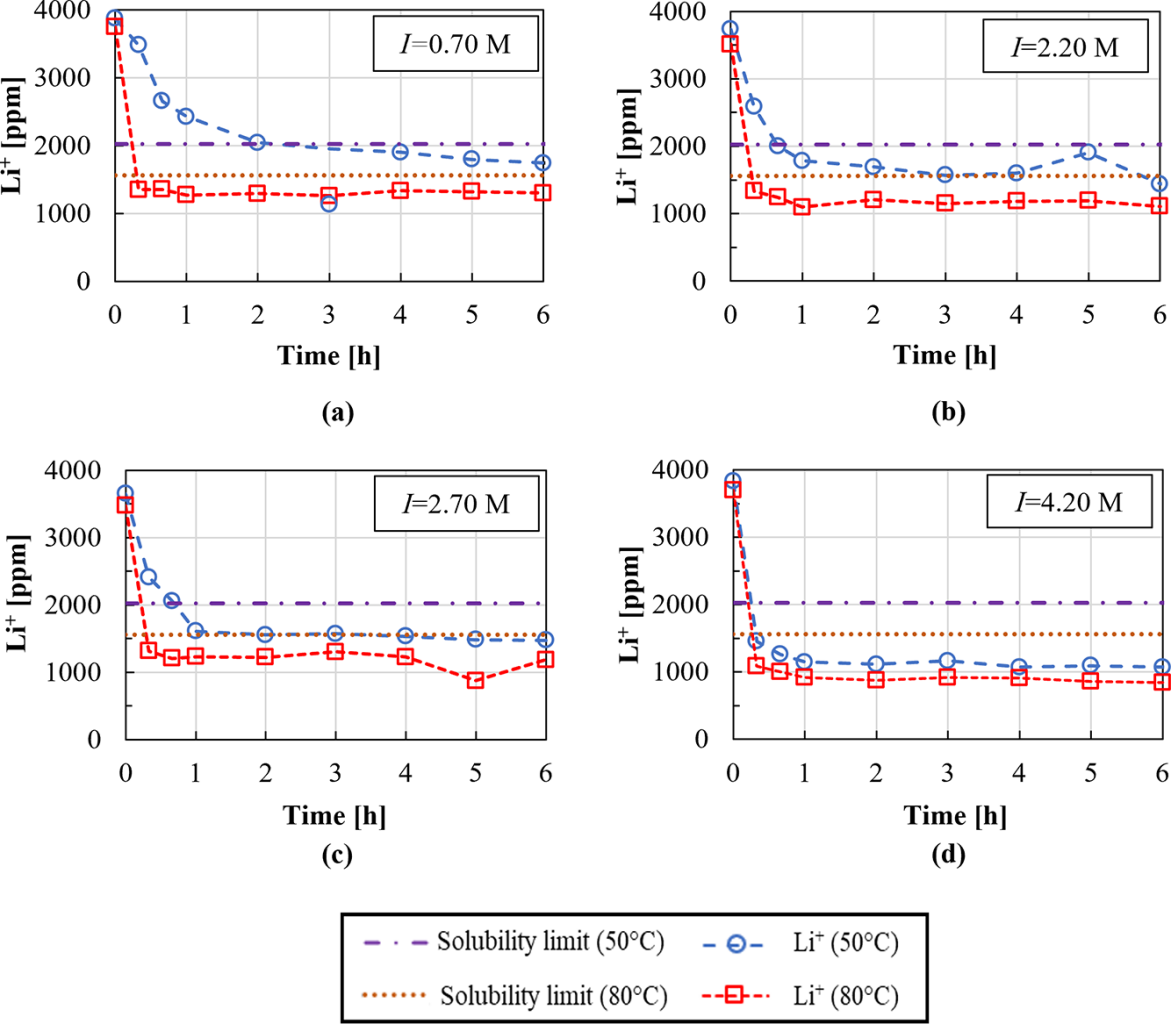
Lithium concentration over time at 50 °C (dashed lines with circle symbols) and 80 °C (dotted lines with square symbols): (a) in pure LiCl (*I* = 0.70 M) solution and in 0.70 M LiCl solutions adding (b) 1.5 M KCl (*I* = 2.20 M), (c) 2.0 M NaCl (*I* = 2.70 M), and (d) 2.0 M NaCl and 1.5 M KCl (*I* = 4.20 M). Stirring speed = 300 rpm, CO_3_^2–^/Li^+^ ratio = 1, and Na_2_CO_3_ solution flow rate = 10 mL/min.

Li^+^ concentration evolution over time during the precipitation tests is shown in Figure 5.

A final Li^+^ concentration of ∼15% lower than the ideal solubility value is obtained in pure LiCl solutions at 50 and 80 °C (Figure 5a), thanks to the over-stoichiometric amount of CO_3_^2–^. Note that, in Figure 5a, the experimental point determined at 3 h was likely affected by some measurements errors, for example, a possible wrong dilution before analysis; therefore, it was excluded from the interpolated Li concentration trend. When other ions are present, Li concentration further decreases reaching values ∼25% lower than the ideal solubility value for the case of single K^+^ or Na^+^ ions added (Figure 5b,c). This is induced by the ion salting-out effect between Na^+^, K^+^, and Li^+^ ions that leads to a Li_2_CO_3_ solubility decrease. The lower Li_2_CO_3_ solubility induces a higher precipitated Li_2_CO_3_ mass (higher reaction yield) and, in turn, a lower final Li^+^ concentration in the solutions. The observed results are in accordance with data reported in the literature^29,30^ and better discussed in the Supporting Information. Finally, the simultaneous presence of Na^+^ and K^+^ ions causes a considerable drop in Li^+^ concentration, in the range of ∼50–60% lower than the ideal solubility at 50 and 80 °C (Figure 5d). It should be also observed that Li_2_CO_3_(s) precipitation is more than two times faster at 80 °C (∼20 min) than that at 50 °C (∼1 h), but with high ionic strength solutions, the kinetics of the precipitation at medium temperatures seems to be enhanced and the precipitation occurs at a comparable time.

**Figure 6:**
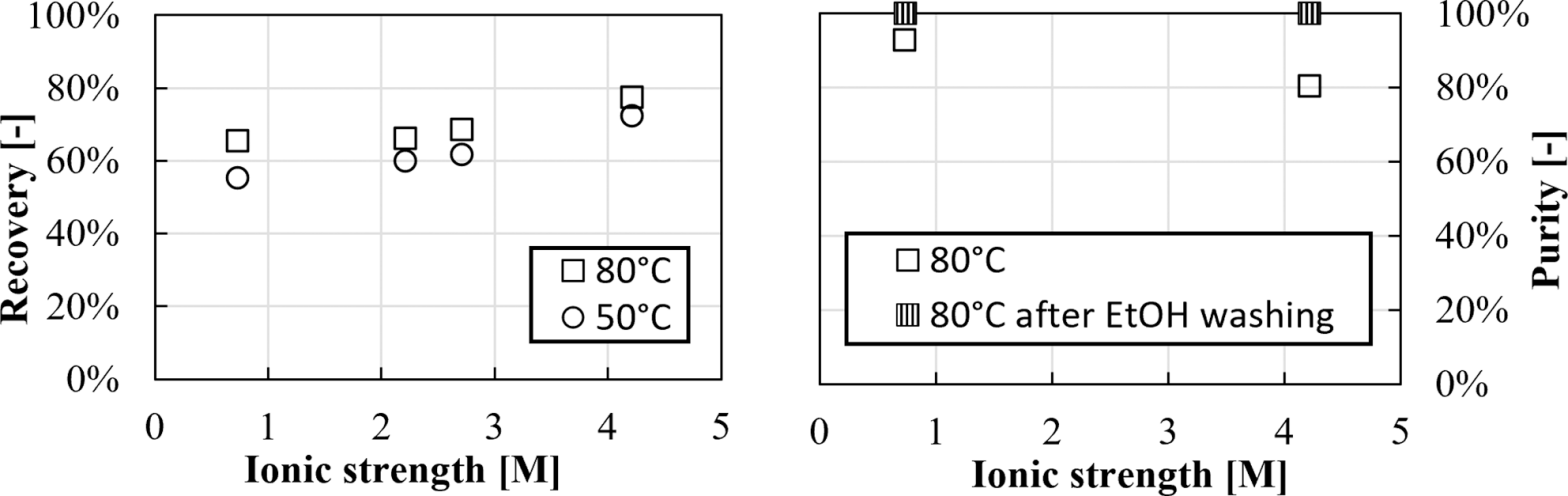
Recovery and purity of Li_2_CO_3_(s) as a function of ionic strength for Li_2_CO_3_ precipitation experiments performed with and without the presence of Na^+^ and K^+^ ions in solution.

Figure 6 shows the Li recovery and purity as a function of solution ionic strength and temperature. For the tests at 80 °C at 0.70 and 4.20 M ionic strength, also recovery and solid purity after the EtOH washing step are reported.

As already commented, the salting-out effect leads to a higher reaction yield, with a Li^+^ recovery increase passing from values around 55 and 65%, for pure LiCl solution, to 72 and 77% (at 50 and 80 °C, respectively), in the case of simultaneous dissolution of Na^+^ and K^+^ ions. Purity of solids obtained in the two extreme cases was analyzed, showing a significant drop from ∼95 to ∼80%, due to the presence of Na^+^ and K^+^ salts in the liquor entrapped in the crystals and on the surface of the crystals, which precipitate during the drying process. However, Li_2_CO_3_(s) purities can be enhanced up to 100% via solid washing with ethanol, causing, on the other hand, a loss of product, resulting in an equivalent reduction of Li recovery from 77 to 57% at 80 °C.

#### 3.1.3 Influence of Divalent Cations: Ca^2+^, Mg^2+^ and Sr^2+^

The influence of dissolved divalent cations, that is, Mg^2+^, Ca^2+^, and Sr^2+^ ions, in LiCl solutions on the Li_2_CO_3_(s) precipitation process was studied. Such ions can form poorly soluble compounds in basic CO_3_^2–^-containing solutions. 0.70 M LiCl solutions were prepared also by dissolving 2.0 M NaCl and 1.5 M KCl to increase solution ionic strength. Also, 0.17 M CaCl_2_, 0.25 M MgCl_2_, and 0.17 M SrCl_2_ salts were added simultaneously and once at time. Details for all the investigated cases are reported in Table S4 in the Supporting Information. Note that all salt concentrations refer to the feed before the addition of Na_2_CO_3_ solution.

**Figure 7:**
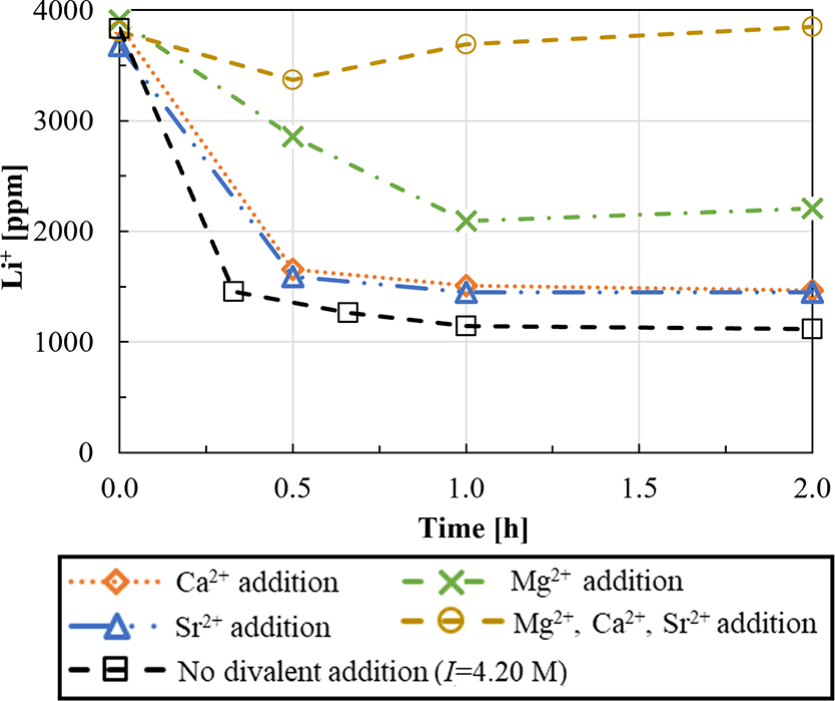
Lithium concentration vs time without any divalent dissolved ions (*I* = 4.20 M, dashed line with square symbols) and with addition of (i) 0.17 M CaCl_2_ (dotted line with rhombus symbols), (ii) 0.25 M MgCl_2_ (dashed lines with cross-symbols), (iii) 0.17 M SrCl_2_ (dot-dashed lines with triangle symbols), and (iv) 0.17 M CaCl_2_ + 0.25 M MgCl_2_ + 0.17 M SrCl_2_ (dashed lines with circle symbols). Stirring speed = 300 rpm, CO_3_^2–^/Li^+^ ratio = 1, and Na_2_CO_3_ solution flow rate = 10 mL/min. *T* = 50 °C.

All precipitation tests were carried out at 50 °C with a stirring velocity of 300 rpm and a double excess of a 2.0 M Na_2_CO_3_ solution (CO_3_^2–^/Li^+^ ratio of 1), fed at a flow rate of 10 mL/min. Figure 7 shows Li^+^ concentration, after the complete addition of Na_2_CO_3_ solutions, over time for the cases reported in Table S4.

From Figure 7, in the presence of Ca^2+^ and Sr^2+^ single salts, a final 37% higher lithium concentration, ∼1500 mg/L, is attained with respect to that in the case of no divalent ion addition. An even higher Li^+^ concentration, that is, ∼2000 mg/L (which means much lower recovery, ∼45%), is measured in the presence of Mg^2+^ salt. This can be attributed to the different influences of divalent ions on the Li_2_CO_3_ solubility. Ma et al.^31^ reported a Li_2_CO_3_ solubility decrease in the presence of dissolved Mg^2+^ ions, although to a lesser extent with respect to monovalent ion cases. Therefore, it can be expected that also Ca^2+^ and Sr^2+^ reduce Li_2_CO_3_ solubility, thus inducing a decrease in the final Li^+^ concentration in the solution. The higher final Li^+^ concentration in the Mg^2+^ case, however, can be attributed to the greater initial Mg^2+^ concentration and a possible superior influence of Ca^2+^ and Sr^2+^ on Li_2_CO_3_ solubility. In all cases, it must stress that, Ca^2+^, Sr^2+^, and Mg^2+^ carbonate compounds have a low solubility that likely causes a CO_3_^2–^ consumption. This is also confirmed by results presented by King et al.^32^ that detected traces of CaCO_3_ and MgCO_3_ in Li_2_CO_3_ compounds precipitated from Li solutions containing 0.033 M Ca^2+^ and Mg^2+^. The simultaneous presence of the three interfering cations (Ca^2+^, Sr^2+^, and Mg^2+^) inhibits Li_2_CO_3_ precipitation, most likely due to the complete consumption of carbonates ions by precipitation of the added divalent cation salts.

**Figure 8:**
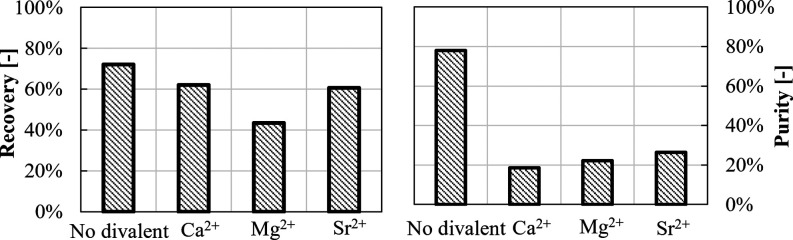
Recovery and purity for Li_2_CO_3_ precipitation experiments in the presence of divalent cations in high-ionic strength solutions. No recovery was calculated in the simultaneous presence of Ca^2+^, Sr^2+^, and Mg^2+^ since no precipitation occurred.

Li^+^ recovery and purity values in the presence of divalent cations are shown in Figure 8.

**Figure 9:**
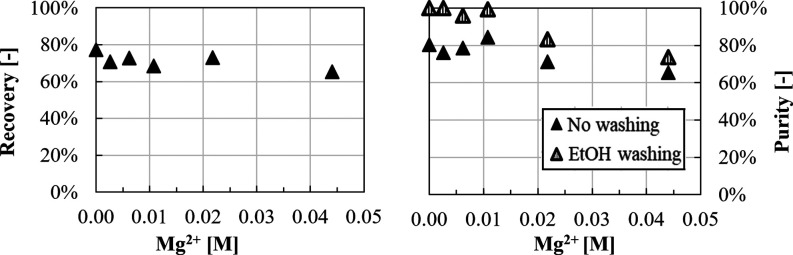
Recovery and purity as a function of initial magnesium concentration. LiCl solutions of 0.70 M with added salts: 2.0 M NaCl and 1.5 M KCl. *T* = 80 °C. Stirring speed = 300 rpm, CO_3_^2–^/Li^+^ ratio = 1, and Na_2_CO_3_ solution flow rate = 10 mL/min.

As already commented in Figure 6, Li^+^ recovery can reach a value around 70% for high-ionic strength solutions without any divalent ions. Here, the presence of divalent ions causes a Li^+^ recovery decrease to ∼60 and ∼40% in the case of Ca^2+^ or Sr^2+^ and Mg^2+^ ions, respectively. Li^+^ recovery is totally inhibited in the simultaneous presence of all three divalent salts (no recovery). The negative impact of the presence of divalent ions can be also observed on the low Li_2_CO_3_(s) purity, never exceeding 28% due to the co-precipitation of other carbonate compounds. Due to the considerable impact of divalent ion presence on the Li_2_CO_3_ precipitation process, the influence of Mg^2+^ concentration was further investigated considering only Mg^2+^ traces, which are likely to be present in the Li-MFCDI eluates of the actual SEArcularMINE treatment chain. In this case, precipitation was carried out at 80 °C (again, to focus on the expected condition in the actual treatment chain) by varying the Mg^2+^ concentration from ∼0.003 to ∼0.044 M. For the sake of brevity, only Li recovery and purity are reported in Figure 9 as functions of the initial Mg concentration.

In this case, Li^+^ recovery values are close to ∼70% for all Mg^2+^ concentrations, thanks to the higher employed temperature; although, also in this case, they result in a lower recovery than that obtained with monovalent salts solutions (78%). A non-monotonic Li^+^ purity trend is observed with increasing Mg^2+^ concentration. Specifically, the purity increases from ∼80 to ∼90% up to a Mg^2+^ concentration of 0.01 M, which further decreases at higher Mg^2+^ concentrations. Purity decreases to values around 60% even at a low Mg concentration of 0.044 M, indicating that the presence of Mg^2+^ ions represents a crucial issue in Li_2_CO_3_ recovery processes from Mg^2+^-containing sources (a better combined strategy to by-pass this issue will be presented in Section 3.2.3). After the purification step with ethanol, purity values increase, leading to an almost monotonical decreasing trend, when increasing Mg^2+^ concentration. However, for higher Mg^2+^ concentrations, the washing step was unable to reach the 100% purity observed in the previous tests, thus again indicating the dramatic influence of Mg salts co-precipitation on the product purity. Also in this case, a loss of product is observed, resulting in an equivalent reduction of Li recovery from 70 to 57%.

#### 3.1.4 Influence of Sulfates and Bromides on Li_2_CO_3_(s) Precipitation

The influence of sulfate and bromide anions on the Li_2_CO_3_(s) precipitation was studied by preparing six different solutions containing 0.70 M LiCl plus

- 1.4 M Na_2_SO_4_ (*I* = 4.90 M)
- 1.0 M KCl and 1.4 M Na_2_SO_4_ (*I* = 5.90 M)
- 1.0 M NaBr (*I* = 1.70 M)
- 1.1 M KCl and 1.0 M NaBr (*I* = 2.80 M).

**Figure 10:**
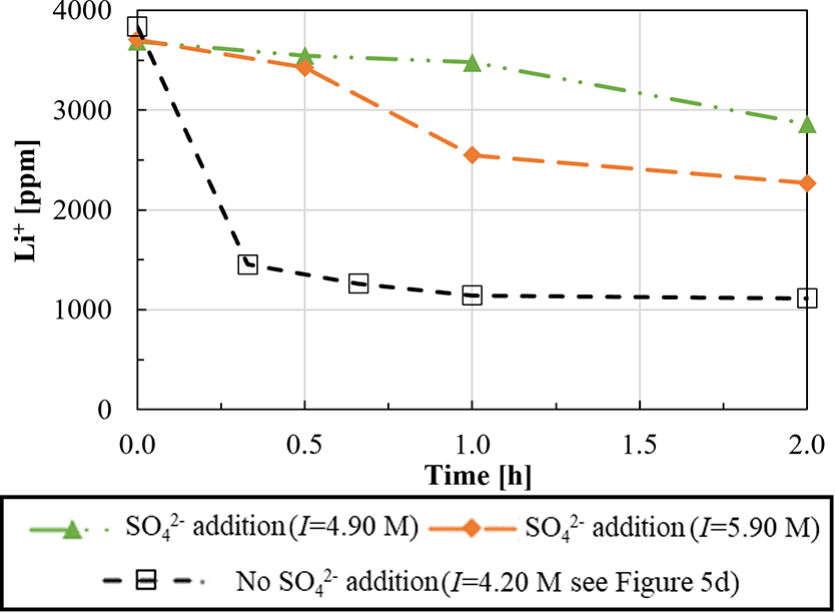
Li^+^ concentration over time in a 0.70 M LiCl solution containing (i) 1.4 M Na_2_SO_4_ (dashed lines with rhombus symbols, *I* = 4.90 M), (ii) 1.4 M Na_2_SO_4_ and 1.0 M KCl (*I* = 5.90 M, dot-dashed lines with triangle symbols), and (iii) without Na_2_SO_4_ (*I* = 4.20 M, dashed lines with square symbols, see Figure 5d). *T* = 50 °C. Stirring speed = 300 rpm, CO_3_^2–^/Li^+^ ratio = 1, and Na_2_CO_3_ solution flow rate = 10 mL/min.

**Figure 11:**
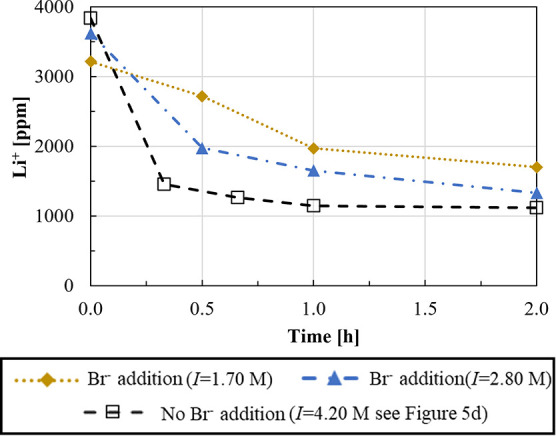
Li^+^ concentration over time in a 0.70 M LiCl solution containing (i) 1.0 M NaBr (*I* = 1.70 M, dotted lines with rhombus symbols), (ii) 1.1 M KCl and 1.0 M NaBr (*I* = 2.80 M, dot-dashed lines with triangle symbols), and (iii) without NaBr (*I* = 4.20 M, dashed lines with square symbols, see Figure 5d). *T* = 50 °C, stirring speed = 300 rpm, CO_3_^2–^/Li^+^ ratio = 1, and Na_2_CO_3_ solution flow rate = 10 mL/min.

Note that all salt concentrations refer to solutions before Na_2_CO_3_ solution addition. All precipitation tests were carried out at 50 °C with a stirring velocity of 300 rpm and a double excess of a 2.0 M Na_2_CO_3_ solution (CO_3_^2–^/Li^+^ ratio of 1), fed at a flow rate of 10 mL/min. The Li^+^ concentration trends during the precipitation time in the presence of sulfate and bromide ions are shown in Figures 10 and 11, respectively.

From Figure 10, it can be seen that the Li_2_CO_3_ precipitation rate considerably decreases in the presence of sulfate, in accordance with the reported delaying effect of sulfate ions on Li_2_CO_3_(s) nucleation.^32^ The delaying effect is reduced in high-ionic strength solutions, although no precipitation occurs within the experiments time; thus, no recovery and purity were calculated. It is worth noting that the dissolution of Na_2_SO_4_ salts also causes a salting-in effect that, in turn, leads to a Li_2_CO_3_ solubility increase, affecting the overall precipitation process.

Figure 11 shows the Li^+^ concentration trend in the presence of Br^–^. It can be observed that Br^–^ ions do not significantly affect the Li precipitation since similar concentration trends as those for pure LiCl solutions, see Figure 5a, are obtained. Furthermore, in the presence of KCl salt (*I* = 2.80 M), a final Li^+^ concentration close to that in high-ionic strength solution without dissolved Br^–^ ions (*I* = 4.20 M) is observed.

**Figure 12:**
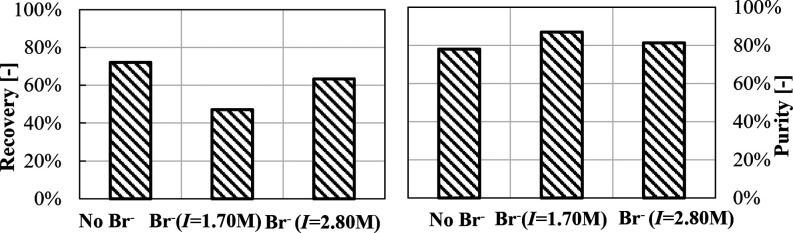
Lithium recovery and purity for Li_2_CO_3_(s) precipitation experiments in the presence of Br ions.

Figure 12 shows purity and recovery values for Li_2_CO_3_ solids precipitated from solutions containing Br^–^ ions.

A Li recovery of ∼47% is found in the presence of Br^–^ ions, which increases up to 63% in higher-ionic strength solutions, almost as that in the case with no Br^–^ ions (72%, see Figure 5d). Similar purity values are observed in high-ionic strength solutions with and without Br^–^ ions (∼80%).

### 3.2 Lithium Precipitation with NaOH/CO_2_(g)

The recovery of Li^+^ using a NaOH solution and CO_2_ gas insufflation represents a promising and environmentally friendly strategy for Li_2_CO_3_(s) production and CO_2_ capture. The influence of several operating parameters was investigated on lithium recovery adopting such a precipitation strategy, namely, (i) the influence of the OH^–^/Li^+^ ratio, (ii) the influence of temperature and solution ionic strength, and (iii) the influence of dissolved magnesium ions.

#### 3.2.1 Influence of the OH^–^/Li^+^ Ratio

The influence of the OH^–^/Li^+^ ratio on Li_2_CO_3_(s) precipitation in a gas–liquid system was investigated within a OH^–^/Li^+^ mole ratio between 1 and 4. Experiments were conducted at 80 °C employing different 8.0 M NaOH volume solutions. The solution was steadily stirred at 300 rpm, and CO_2_ gas was fed at a flow rate of ∼4.5 L/h. Details of the reacting solutions are reported in Table S5 in the Supporting Information.

**Figure 13:**
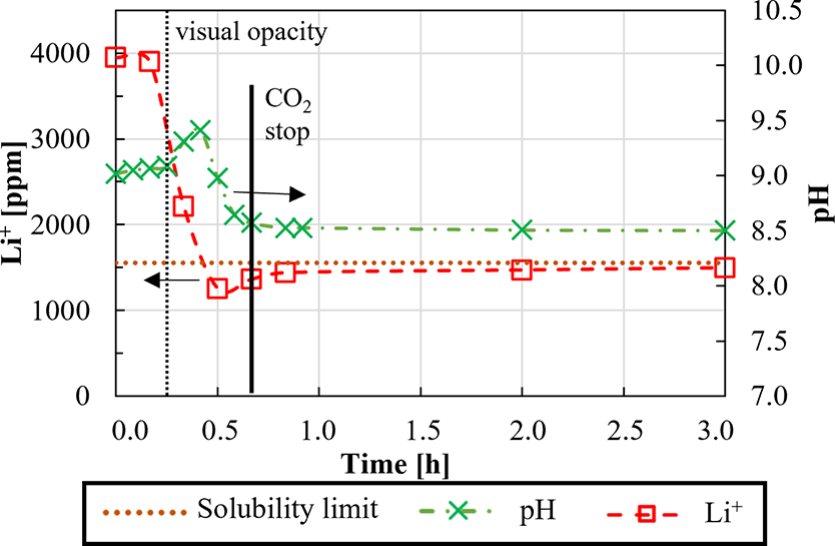
Lithium concentration (dashed lines with square symbols) and pH (dot-dashed lines with cross-symbols) versus time for a OH^–^/Li^+^ = 2. Li^+^ initial concentration after NaOH solution addition of ∼3900 ppm, *T* = 80 °C, and stirring speed = 300 rpm. CO_2_ flow rate ≈ 4.5 L/h.

In addition to the Li^+^ concentration variation along time, Figure 13 reports also the solution pH and indications on the visual opacity threshold observed during the experiment, thus allowing a more phenomenological interpretation of the experiment.

For the sake of brevity, such trends are reported only for the OH^–^/Li^+^ ratio of 2, although similar considerations hold for the other cases.

Starting from time = 0, after the addition of the alkaline reactant and starting insufflating CO_2_, the solution pH increases slightly from 9.0 to 9.1 until the solution becomes turbid, indicating that Li_2_CO_3_ precipitation has started. Then, pH increases up to ∼9.4 to further sharply decrease to 8.5. At such a pH value, CO_2_(g) is stopped (40 min) to prevent a pH decrease, causing Li_2_CO_3_ “re-carbonation” (see the Supporting Information for further details). As for the pH, the Li^+^ concentration remains almost constant until the solution becomes turbid to suddenly drop to a value of ∼1300 ppm after 30 min, and then, it slightly increases again to a final concentration of ∼1450 ppm caused by very slight re-carbonation of Li_2_CO_3_. No further concentration variation is observed after CO_2_ interruption.

**Figure 14:**
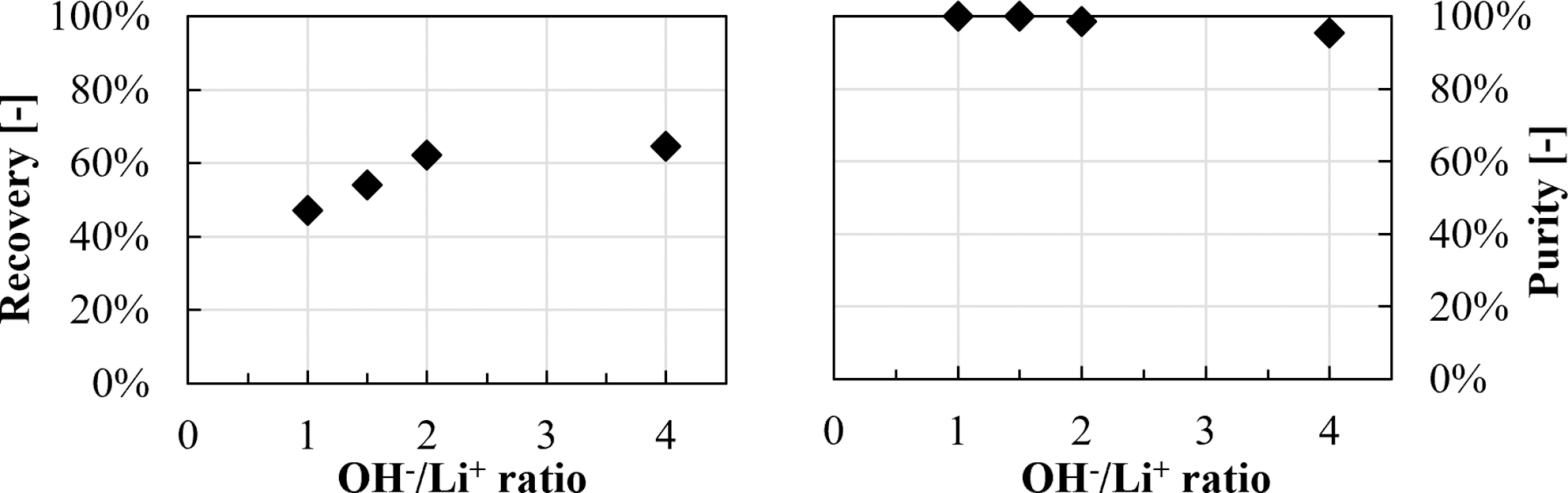
Li_2_CO_3_ recovery and purity at different OH^–^/Li^+^ ratios.

The recovery and purity as a function of the OH^–^/Li^+^ ratio are reported in Figure 14.

The Li^+^ recovery increases from ∼45 to ∼65%, increasing the OH^–^/Li^+^ ratio from 1 to 4, while purity nearly reaches 100% in all cases.

#### 3.2.2 Influence of Solution Ionic Strength and Temperature

**Figure 15:**
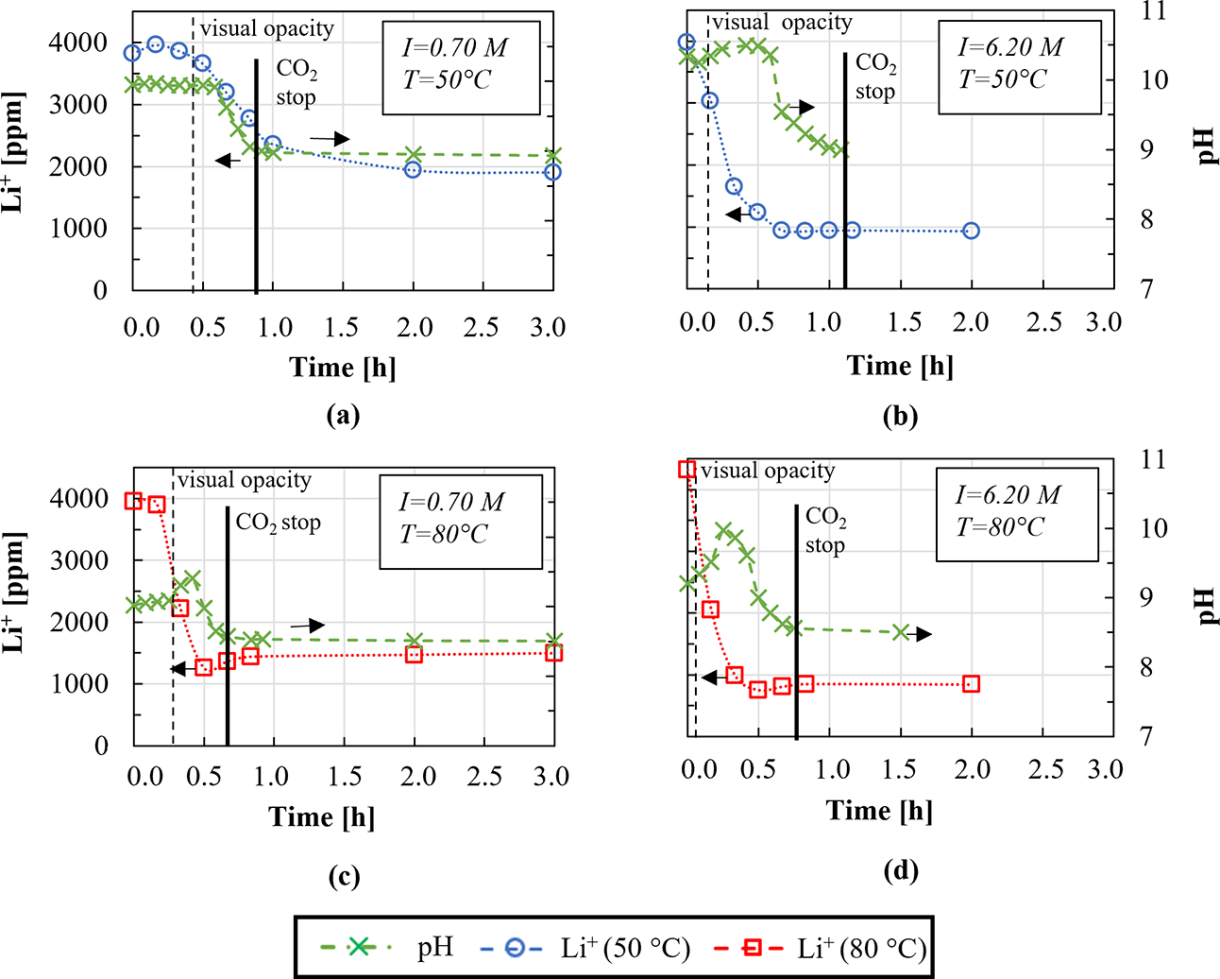
Lithium concentration (dotted lines with circles and square symbols) and pH (dashed lines with cross-symbols) as a function of experimental time. Experiments were performed at 50 (a,b) and 80 °C (c, d) employing 0.70 M LiCl solutions without the addition of further ions (a,c) and adding 2.2 M NaCl, 3.3 M KCl (b,d). Stirring speed of 300 rpm and the OH^–^/Li^+^ ratio of 2. The CO_2_ flow rate of (a,c) 1.8 and (b,d) 4.5 L/h.

To study the influence of temperature and ionic strength on the Li_2_CO_3_ precipitation using NaOH solution and CO_2_ insufflation, four tests were carried out. Specifically, starting from the reference conditions presented above, an additional precipitation test was performed at 50 °C using pure 0.70 M Li^+^ solutions, and tests at 50 and 80 °C were performed adding 2.2 M NaCl and 3.3 M KCl to increase the solution ionic strength up to 6.20 M. Salt concentrations refer to solutions before NaOH solution addition. Solutions were steadily stirred at 300 rpm. In all the experiments a OH^–^/Li^+^ ratio of 2 was used. The CO_2_ flow rate was 1.8 and 4.5 L/h at 50 and 80 °C, respectively. Figure 15 reports solution pH and Li concentrations during the experiment.

As can be seen in Figure 15, solution pH values remain almost constant until the solution becomes turbid. After turbidity detection, pH increases for ∼30 min to further decrease until CO_2_ is stopped. Only in the case of low-ionic strength solutions at 50 °C, pH remains constant after turbidity detection and decreases after ∼20 min. After CO_2_ insufflation interruption, solution pH settles to final values of 8.5 and 9.0 at 80 and 50 °C, respectively. Sun et al.^23^ reported pH values of 9.0–9.5 when performing Li_2_CO_3_ precipitation from 14,000 ppm LiCl solution at 20 °C. Conversely, Han et al.^19^ measured a lower pH value of 8.0 at 25 and 50 °C using, however, a staring 20,000 ppm Li_2_SO_4_ solution.

In all the experiments, Li^+^ concentration remains almost constant until the solution turbidity detection to further decrease sharply. In the case of low-ionic strength solutions, final Li^+^ concentration values of ∼1500 ppm are reached, while, in high-ionic strength solution environment, the final Li^+^ concentration decreases up to 50%.

From Figure 15, it is also noted that Li_2_CO_3_ precipitation is faster at 80 °C, but it is even faster in high-ionic strength solutions, where almost no induction time is recorded.

**Figure 16:**
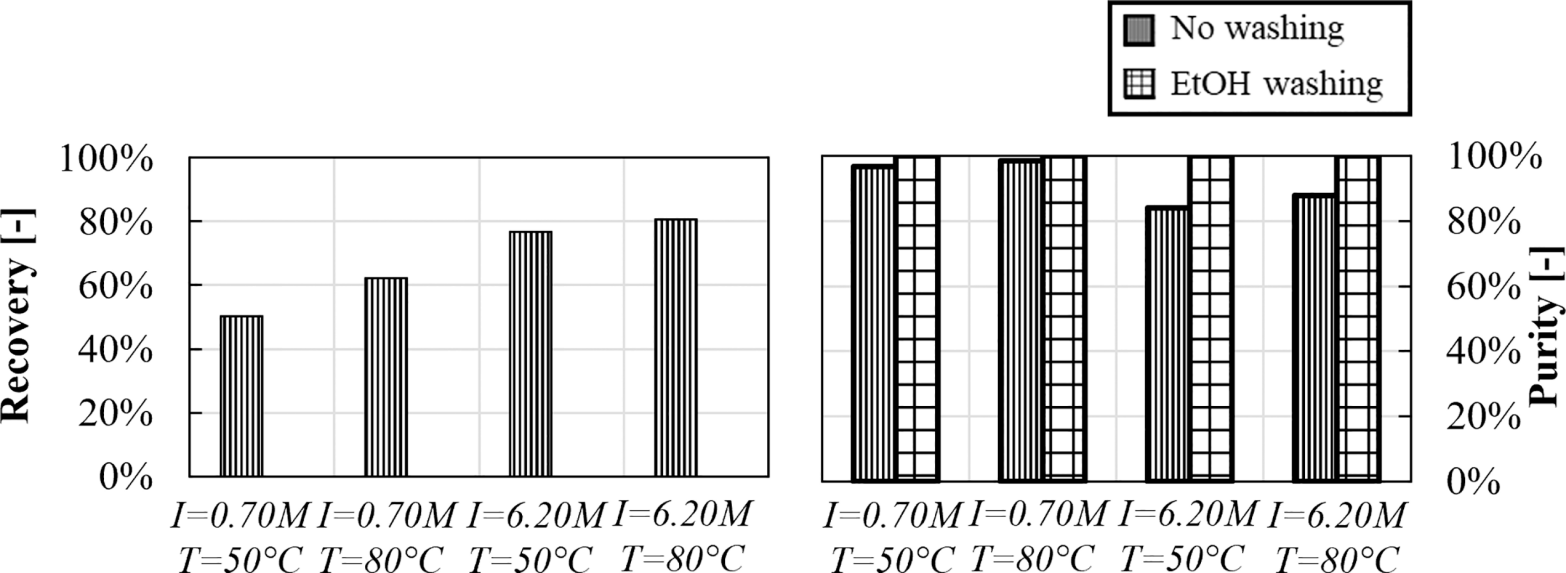
Recovery and purity for Li_2_CO_3_ precipitation experiments from a gas–liquid system in LiCl solutions with high and low ionic strength at 50 and 80 °C.

Li^+^ recovery and purity are reported in Figure 16, along with purity after ethanol washing.

Li^+^ recovery increases from ∼50 to ∼60% with increasing temperature from 50 to 80 °C. Higher recovery values are measured in high-ionic strength solutions, that is, from 60 to 80% at 80 °C. Purity values are almost 100% in low-ionic strength solutions, but significantly decrease to ∼85% in high-ionic strength ones. Purity can be enhanced up to 100% by ethanol washing, causing, however, recovery losses, for example, from ∼80 to ∼60% in high-ionic strength solutions at 80 °C. Results are in accordance with the discussed influence of monovalent ions on the Li_2_CO_3_ solubility, presented in Section 3.1.1.

#### 3.2.3 Influence of Magnesium Concentration on Li_2_CO_3_(s) Precipitation

As discussed in Section 3.1.3, it is expected that LiCl solution from real bitterns may contain traces of Mg^2+^, even after Mg^2+^ removal and selective Li extraction in the abovementioned SEArcularMINE process. Thus, the detrimental influence of Mg^2+^ traces in Li^+^ feed solutions was also studied in the case of NaOH + CO_2_ precipitation, considering a possible Mg^2+^ concentration range from 0 to 0.2 M. Since Li_2_CO_3_(s) forms after the addition of NaOH solutions and the insufflation of CO_2_, the possibility of performing the precipitation into a two-step process was investigated, with (i) first basification of the solution (OH^–^ addition stage), in which Mg(OH)_2_ solids precipitated and were then filtered out and (ii) carbonization (CO_2_ insufflation stage) of the filtered solution for lithium carbonate precipitation. For comparison purposes, for the case of a LiCl solution containing a Mg^2+^ concentration of 0.08 M only, Li_2_CO_3_(s) precipitation was performed with and without filtration. All experiments were performed adding 1.8 M NaCl and 3.0 M KCl to increase ionic strength of the solution. Salt concentrations refer to solutions before NaOH addition. Temperature was kept at 50 °C, and solutions were stirred at 300 rpm. The CO_2_ flow rate was ≈4.0 L/h.

**Figure 17:**
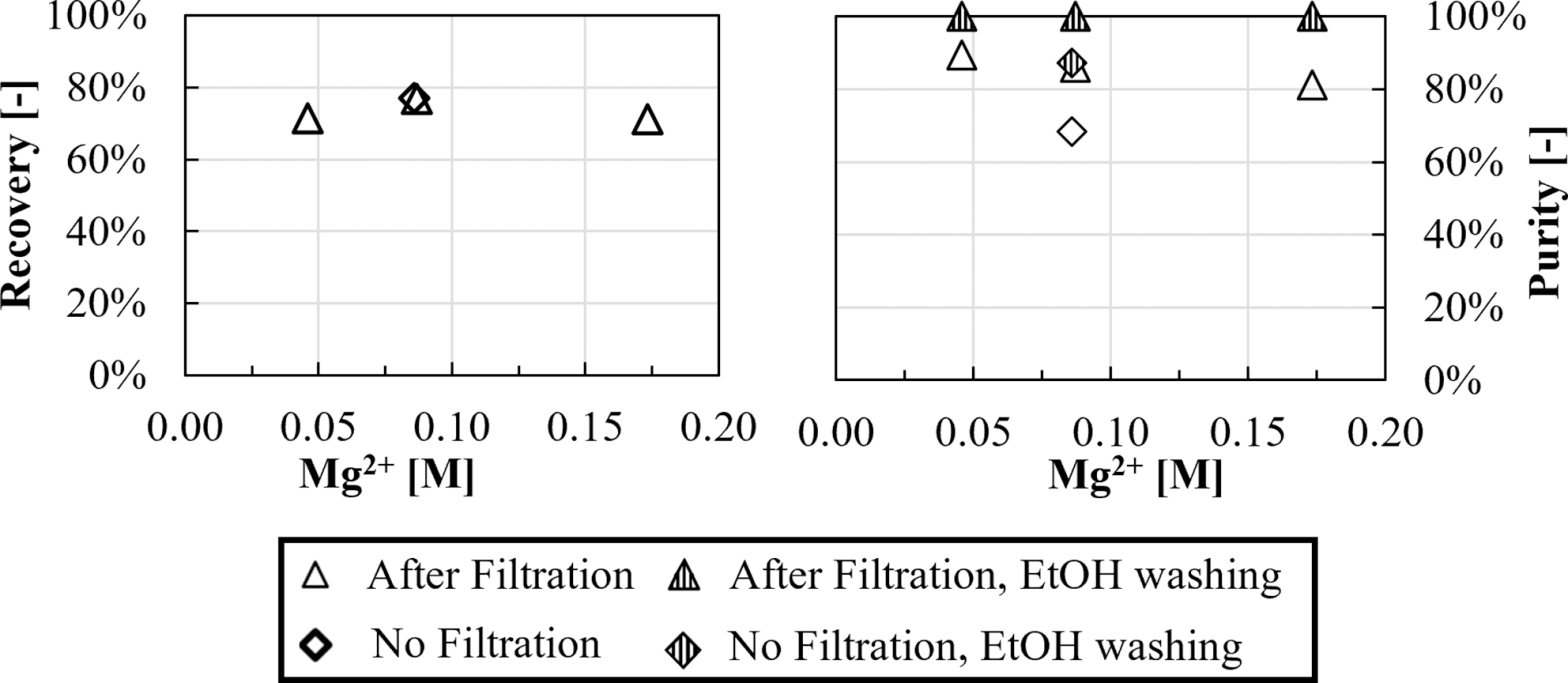
Recovery and purity over magnesium concentration in 0.70 M LiCl, a OH^–^/Li^+^ ratio of 2, 3.0 M KCl and 1.8 M NaCl. *T* = 50 °C, a stirring speed of 300 rpm, and a CO_2_ flow rate of ≈4.0 L/h.

Li^+^ recovery and purity values as a function of Mg^2+^ concentration are reported in Figure 17.

Similar final Li(l) concentrations of ∼800 ppm were measured in all tests leading to recovery values of about ∼70–75%. Purity decreases with increasing Mg^2+^ concentration from 90% (0.04 M Mg^2+^) to 80% (0.18 M Mg^2+^) caused by the co-precipitation of Mg(OH)_2_(s) and MgCO_3_. Purities can be enhanced up to 100% by applying ethanol washing. It is worth noting that, when the basification step (in which Mg(OH)_2_ precipitates) is not followed by filtration (case at Mg^2+^ 0.08 M), a similar recovery of ∼75% is observed, while purity considerably drops from ∼87 to ∼68%. In this case, the ethanol washing step is not able to increase the purity above 90%, as it was also reported in Section 3.1.3. Such a result demonstrates that Mg(OH)_2_ precipitation and filtration before CO_2_ insufflation and Li_2_CO_3_ precipitation can be employed as a promising approach to first eliminate Mg^2+^ content in LiCl solutions and then obtain Li_2_CO_3_ solids with high purity (∼90%) and recovery (∼70%).

### 3.3 Comparison between Li^+^ Precipitation Using Na_2_CO_3_ and NaOH/CO_2_ Insufflation

The precipitation of Li_2_CO_3_ in LiCl solutions using either Na_2_CO_3_ or NaOH solutions and CO_2_(g) insufflation alternatives was extensively addressed in Sections 3.1 and 3.2. Table 1 reports a comparison between recovery and purity results obtained from the two precipitation approaches: (i) in the case of a double excess of the precipitants at 80 °C (reference case, see Sections 3.1.1 and 3.2.1), (ii) at a low temperature of 50 °C (see Figures 5a and 15a), (iii) in the presence of high-ionic strength solutions at 80 °C (Figures 5d and 15d), and (iv) in LiCl solution in the presence of 0.04 M Mg^2+^ concentration at 80 °C (Figures 9 and 17).

**Table 1:** Comparison between Li^+^ Recovery and Purity Obtained Using Either Na_2_CO_3_ or NaOH Solution and CO_2_(g) Insufflation Precipitation Routes

	precipitation method	*T* [°C]	recovery [%]	purity [%]	equivalent recovery after EtOH washing [%]	purity after EtOH washing [ %]
reference case	Na_2_CO_3_, (CO_3_^2–^/Li^+^ = 1)	80	∼62	∼95		
	NaOH & CO_2_(g), (OH^–^/Li^+^ = 2)	80	∼60	∼99		
low temperature	Na_2_CO_3_	50	∼55	∼94		
	NaOH & CO_2_(g)	50	∼50	∼97	∼45	∼100
high ionic strength	Na_2_CO_3_	80	∼77	∼80	∼53	∼100
	NaOH & CO_2_(g)	80	∼80	∼90	∼60	∼100
0.04 M Mg concentration	Na_2_CO_3_	80	∼65	∼65	∼50	∼75
	NaOH & CO_2_(g) after filtration	80	∼70	∼90	∼60	∼100

From Table 1, it can be observed that temperature is a crucial parameter for Li recovery. The lowest recovery values of ∼50% are, in fact, achieved at 50 °C. Li recovery can be increased by using high-ionic strength solutions, reaching the highest measured recovery value of 80% when using NaOH and CO_2_ insufflation at 80 °C. On the other hand, purity values range from 65 to 90% in high-ionic strength solution or in the presence of Mg ions. Conversely, solids produced from pure LiCl solutions exhibit purities higher than 94%. The ethanol washing step allows the production of 100% pure solids, causing, however, a Li^+^ reduction of equivalent recovery that ranges from 45 to 63%. Results also highlight that the Li_2_CO_3_(s) precipitation using NaOH solutions and CO_2_ insufflation can be pursued as a promising alternative for the simultaneously recovery of Li^+^ and CO_2_ capture since results are similar to those obtained using the classical Na_2_CO_3_ precipitant, especially due to the option of enhancing the purity by a simple filtration step without losing the product in the presence of divalent ions.

Li_2_CO_3_ reaction times can also be compared between results of the two precipitation approaches, see Figures 5 and 15. Specifically, precipitation times were selected when Li^+^ concentrations did not vary more than 10% in two consecutive measurements. Table 2 reports a comparison between the precipitation times at 50 and 80 °C in LiCl solutions with and without salt addition.

Li_2_CO_3_ precipitation is faster at 80 °C, showing similar reaction times of about 50–60 min for both precipitation approaches. Similar reaction times are also observed in high-ionic strength solutions. At 50 °C, the precipitation is faster in gas–liquid systems (120 min against 300 min for Na_2_CO_3_), while it is more than two times faster in high-ionic strength solutions.

**Table 2:** Comparison of the Reaction Times during Li_2_CO_3_ Precipitation Tests

temperature [°C]	solution	Na_2_CO_3_ [min]	NaOH and CO_2_(g) [min]
50	pure LiCl	300	120
	high ionic strength	60	60
80	pure LiCl	60	60
	high ionic strength	60	50

### 3.4 Process Performance Comparison with the State of Art

For the sake of comparison with the state of art, an overview of recent literature studies is reported below for the Li_2_CO_3_ precipitation from Li brines, followed by a comparative table with the present work’s best identified scenario.

An et al.^33^ presented a two-stage Li extraction process from Uyuni Salar brine (Bolivia) containing 700–900 mg/L Li^+^ and 15,000–18,000 mg/L Mg^2+^, among the other ions. First Mg^2+^, Ca^2+^, and sulfates were removed by precipitation using lime and sodium oxalate. Then, the purified brine was concentrated 30 folds by evaporation, reaching a final Li^+^ concentration of 20,000 mg/L. The concentrated brine also contained 56,000, 52,000, <0.05, 350, and 20,000 mg/L concentrations of Na^+^, K^+^, Ca^2+^, Mg^2+^, and SO_4_^2–^, respectively. Li_2_CO_3_ precipitation was performed at 80–90 °C by the addition of Na_2_CO_3_. Li_2_CO_3_ solid purity was higher than 99.55%, after employing hot-water washing, while the recovery was estimated to be higher than 90%. Jiang et al.^34^ investigated the production of Li_2_CO_3_ from lithium brines adopting a laboratory-scale electrodialysis system. A synthetic brine was prepared to mimic the ion concentration in Zabuye lake brines (China) that contain a Li^+^ concentration of 879 mg/L. The brine was first treated with Na_2_CO_3_ to reduce Ca^2+^ and Mg^2+^. Afterward, a conventional electrodialysis process was employed to increase the Li^+^ concentration up to 3485 mg/L. The concentrated solution had also 7319, 5.3, and 37 mg/L concentrations of Na^+^, Ca^2+^ and Mg^2+^. After Li_2_CO_3_ precipitation, a secondary crystallization step was adopted to increase powder purity from 90.33 to 95.30%. Unfortunately, the authors did not provide information regarding Li^+^ recovery. Um and Hirato^35^ studied the recovery of lithium from seawater adopting an adsorption Li^+^ selective step with the manganese oxide adsorbent and a further precipitation step. The obtained brine was treated using NaOH to reduce Ca^2+^ and Mg^2+^. Na_2_CO_3_ solution was added into the Li solution that was concentrated by evaporation at 100 °C, decreasing the solution volume to 67, 53, and 40%. The Li_2_CO_3_ yield varied from 51 to 77%; however, the purity decreased from 99.4 to 98.7%. Xu et al.^36^ developed a two-step process to produce battery-grade lithium carbonate from the Damxungcuo saline lake brine (Tibet). The brine contained 360 mg/L Li^+^, 54,000 mg/L, 7,300 mg/L, and 810 mg/L Na^+^, K^+^, and Mg^2+^, respectively. Li_2_CO_3_ solids were first produced by evaporation of saline lake solutions and then added to the Li brine. Lime milk and H_2_O_2_ were employed to remove insoluble compounds, NaOH was added to deplete Fe species concentration, and oxalic acid was added to remove Mg(OH)_2_ and Na_2_CO_3_ to treat Ca. After purification, industrial-grade Li_2_CO_3_ was obtained that was further treated using CO_2_ and EDTA-Li (lithium 2-carboxyhydrazine-1,1,2-tricarboxylate) at 85 °C to increase its purity up to 99.6% with a recovery of about 84%. Zhao et al.^27^ studied the recovery of lithium carbonate from synthetic lithium chloride solutions using ultrasounds. Lithium sulfate solutions with a Li concentration between 5000 and 25,000 mg/L were obtained from the leachate of the cathode scrap of lithium-ion batteries. The precipitation process was conducted at 70 °C. Na_2_CO_3_ was added at one time, immediately applying ultrasounds. Recovery and purity were compared with those of classical stirred precipitation systems without the use of ultrasounds. Recovery increased adopting ultrasound varying from 45 to 60 and from 70 to 80% for an initial Li^+^ concentration of 5000 and 10,000 mg/L, respectively. Purity also increased using ultrasounds, showing values higher than 98% at such concentrations. Quintero et al.^37^ developed a process for the direct production of magnesium-doped Li_2_CO_3_ solids by direct co-precipitation of Mg(OH)_2_ treating industrial Li-enriched brines. An industrial refined brine from the Albemarle industrial plant (North of Chile) was used with a concentration of 0.030, 1.14, 0.04, 0.02, and 3.22 % wt for Ca^2+^, Mg^2+^, Na^+^, K^+^, and Li^+^, respectively. Ca^2+^ was removed by using oxalate and NaOH solutions. Furthermore, NaOH was added to precipitate the remaining magnesium. Na_2_CO_3_ solution was used at a 1:2 Li^+^ ratio to co-precipitate Li_2_CO_3_. The Li_2_CO_3_ precipitation process occurred with a Li^+^ initial concentration of 30,000 ppm performed at 80 °C. The Li_2_CO_3_/Mg(OH)_2_ solid recovery was 88%.

Table 3 reports a comparison between Li_2_CO_3_ precipitation approaches presented in the literature and the best scenarios addressed in the present work.

**Table 3:** Comparison Between Li_2_CO_3_ Precipitation Approaches Presented in the Literature and the Best Scenarios Addressed in the Present Work

	An et al.^33^	Jiang et al.^34^	Um and Hirato^35^	Xu et al.^36^	Zhao et al.^27^	Quintero et al.^37^	present work
Li solution	Uyuni salar brine (Bolivia)	synthetic	seawater	Damxungcuo saline lake brine (Tibet)	synthetic	Albemarle industrial plant (North of Chile)	synthetic
Li concentration	evaporation (from 700–900 to 20,000 mg/L)	electrodialysis (from 879 to 3485 mg/L)	adsorption and evaporation (from 0.17 mg/L)	lithium seeds in a Li brine of 360 mg/L	5000–25,000 mg/L	3000 mg/L	∼4000 mg/L with high ionic strength
precipitation conditions	80–90 °C Na_2_CO_3_	Na_2_CO_3_	100 °C, Na_2_CO_3_	20–85 °C, Na_2_CO_3_	ultrasounds. 70 °C, Na_2_CO_3_	80 °C, Na_2_CO_3_ double excess	80 °C, NaOH & CO_2_ (g), high ionic strength
Li recovery	expected >90%		51–77%	84%	60–80%	88%	80% (60% after EtOH washing)
Li purity	99.55% after hot water washing	95.3% after secondary crystallization	99.4–98.7%	99.6% after CO_2_ and EDTA–Li	>98%		90% (100% after EtOH washing)

Results indicate how the NaOH and CO_2_ (g) precipitation route conducted at 80 °C in a high-ionic strength Li solution leads to final Li recovery and purity values not too far from those of the other presented approaches in the literature. Specifically, a recovery of 80% is slightly lower than the other reported values, while the purity passes from 90% of the raw precipitated product up to 100% via an ethanol washing step, thus also confirming the need for a purifying step mentioned in most of the literature studies.

## 4 Conclusions

An extensive experimental investigation on lithium carbonate precipitation from moderately concentrated Li-rich brine was presented, with a focus on recovery and solid purity. Li^+^ was precipitated via homogenous and heterogeneous crystallization routes using Na_2_CO_3_ and a gas (CO_2_)–liquid (NaOH–LiCl) system. Numerous parameters affecting the crystallization process were investigated, also mimicking expected scenarios for implementation within the SEArcularMINE valorization chain with real saltworks bitterns, for example, by dissolving monovalent and divalent ions in Li^+^-containing solutions. For the first time, to the best of authors’ knowledge, experimental results were conducted in the case of heterogeneous Li_2_CO_3_(s) precipitations in the presence of added monovalent and divalent ions in the LiCl–NaOH–CO_2_ system.

First, the influence of reaction temperature and solution ionic strength, by addition of other monovalent ions, that is, K^+^ and Na^+^, in the feed LiCl solutions was investigated. Li^+^ recovery varied from 50%, in the case of low-ionic strength solutions using NaOH and CO_2_(g) at 50 °C, to 80%, in high-ionic strength solutions at 80 °C employing both precipitation routes. This was not only due to the higher employed temperature at which Li_2_CO_3_ had a lower solubility but also due to the interaction between Li^+^, Na^+^, and Ca^2+^ ions that caused a further Li_2_CO_3_ solubility decrease (salting-out effect). On the other hand, Li_2_CO_3_(s) purity decreased from ∼95–99 to ∼80–90% due to the higher concentration of other cations, namely, Na^+^ and K^+^. It is interesting to note that higher purities were obtained using NaOH solutions and the CO_2_(g) insufflation precipitation approach.

Li_2_CO_3_(s) precipitation was found to be faster in high-ionic strength solutions, probably induced by the interaction between added cations, where reaction at 50 °C mostly occurred within 60 min, while up to 120 min were needed in low-ionic strength ones. Such a difference was not observed at 80 °C, where the high temperature led to very similar precipitation rates, thus marking a clear influence of the Li_2_CO_3_ solubility on the precipitation process.

Afterward, the influence of divalent cations and anions, namely, Ca^2+^, Sr^2–^, Mg^2+^, Br^–^, and SO_4_^2–^, added in high-ionic strength LiCl feed solutions was analyzed when employing Na_2_CO_3_ precipitant solutions. Only the influence of dissolved Mg^2+^ ions was studied in the case of NaOH and CO_2_(g) insufflation. The addition of Ca^2+^, Sr^2+^, and Br^–^ ions caused a slight decrease in Li^+^ recovery from ∼80 to ∼60% with respect to the case with no divalents. Purity considerably dropped to values of ∼20% in the presence of Ca^2+^ and Sr^2+^ ions, while a negligible variation was observed in the presence of Br^–^ due to the low solubility of carbonate compounds that mostly precipitated together with Li_2_CO_3_ in the presence of Ca^2+^ and Sr^2+^ ions in solution.

SO_4_^2–^ ions dramatically affected the precipitation process, which was totally inhibited for the 2 h of experimental run caused by the increase in Li_2_CO_3_ solubility and the delay effect of SO_4_^2–^ ions on the precipitation process (salting-in effect).

Considering the presence of Mg^2+^ ions, 40% Li^+^ recovery and 20% Li_2_CO_3_(s) purity were obtained with 0.25 M Mg^2+^ using the Na_2_CO_3_ precipitation route. Further experiments with lower Mg^2+^ concentrations, that is, from 0 to 0.05 M, confirmed the high impact of Mg^2+^ on Li_2_CO_3_(s) purity that was ∼60% even at a Mg^2+^ concentration of 0.05 M, caused by the low solubility of Mg carbonate species.

In the case of the NaOH and CO_2_ insufflation precipitation route, a two-step precipitation process was implemented. First, NaOH solution was added, raising the pH and leading to the precipitation of Mg insoluble salts, and then, CO_2_ was insufflated in the filtered solution. The method was found to be very effective: high Li^+^ recovery (∼70%) and high Li_2_CO_3_(s) purity (∼80%) were obtained even starting with a 0.20 M MgCl_2_ solution.

Li_2_CO_3_ (s) purity was successfully enhanced in several cases by employing an ethanol washing step that allowed to reach solid purity values of ∼99% accompanied, however, by a Li loss of about 10–20%.

Overall, the results provide important guidelines for the best choice of operational conditions and process control for industrial scale-up of Li^+^ recovery from relatively low-concentration brines. Specifically, it was demonstrated that precipitation should be performed at a high temperature (80 °C) to decrease Li_2_CO_3_ solubility, thus achieving higher recovery values. NaCl and KCl salts can be employed to increase Li recovery, thanks to the induced salting-out effect. On the other hand, a purity decrease is expected, requiring a further purification step. Divalent ions should be removed before precipitation due to the low solubility of their carbonate and hydroxide compounds that precipitate using both Na_2_CO_3_ and NaOH solutions. Sulfate ions should be reduced as much as possible before precipitation since they cause a Li_2_CO_3_ solubility increase (salting-in) and a kinetic delay effect. In regard to process control, care must be taken for the accurate control of the pH, especially in the case of the NaOH and CO_2_ precipitation route. In this case, CO_2_ insufflation must be blocked before re-carbonation of Li_2_CO_3_. It is worth noting that the NaOH and CO_2_ insufflation precipitation route represents an appealing potential industrial application, as also discussed in Section 3.4, whose performance is going to be demonstrated on a pilot scale, in the second phase of the SEArcularMINE project, treating real Li-rich brines.

## Supporting Information Available

Fundamentals of Li_2_CO_3_(s) precipitation routes by Na_2_CO_3_ or NaOH/CO_2_ addition along with tables reporting details of the experimental tests discussed in the main text (PDF)

## References

[ref1] Bello A. S.; Zouari N.; Da’ana D. A.; Hahladakis J. N.; Al-Ghouti M. A. An Overview of Brine Management: Emerging Desalination Technologies, Life Cycle Assessment, and Metal Recovery Methodologies. J. Environ. Manage. 2021, 288, 112358 10.1016/j.jenvman.2021.112358. 33770726

[ref2] Kumar A.; Naidu G.; Fukuda H.; Du F.; Vigneswaran S.; Drioli E.; Lienhard J. H. Metals Recovery from Seawater Desalination Brines: Technologies, Opportunities, and Challenges. ACS Sustain. Chem. Eng. 2021, 9, 7704–7712. 10.1021/acssuschemeng.1c00785.

[ref3] Al-Absi R. S.; Abu-Dieyeh M.; Al-Ghouti M. A. Brine Management Strategies, Technologies, and Recovery Using Adsorption Processes. Environ. Technol. Innovat. 2021, 22, 101541 10.1016/j.eti.2021.101541.

[ref4] Pramanik B. K.; Nghiem L. D.; Hai F. I. Extraction of Strategically Important Elements from Brines: Constraints and Opportunities. Water Res. 2020, 168, 115149 10.1016/j.watres.2019.115149. 31604175

[ref5] Loganathan P.; Naidu G.; Vigneswaran S. Mining Valuable Minerals from Seawater: A Critical Review. Environ. Sci.: Water Res. Technol. 2017, 3, 37–53. 10.1039/c6ew00268d.

[ref6] Alsabbagh A.; Aljarrah S.; Almahasneh M. Lithium Enrichment Optimization from Dead Sea End Brine by Chemical Precipitation Technique. Miner. Eng. 2021, 170, 107038 10.1016/j.mineng.2021.107038.

[ref7] Meng F.; McNeice J.; Zadeh S. S.; Ghahreman A. Review of Lithium Production and Recovery from Minerals, Brines, and Lithium-Ion Batteries. Miner. Process. Extr. Metall. Rev. 2021, 42, 123–141. 10.1080/08827508.2019.1668387.

[ref8] Ambrose H.; Kendall A. Understanding the Future of Lithium: Part 1, Resource Model. J. Ind. Ecol. 2020, 24, 80–89. 10.1111/jiec.12949.

[ref9] Kavanagh L.; Keohane J.; Garcia Cabellos G. G.; Lloyd A.; Cleary J. Global Lithium Sources-Industrial Use and Future in the Electric Vehicle Industry: A Review. Resources 2018, 7, 57 10.3390/resources7030057.

[ref10] Bonin L.; Deduytsche D.; Wolthers M.; Flexer V.; Rabaey K. Boron Extraction Using Selective Ion Exchange Resins Enables Effective Magnesium Recovery from Lithium Rich Brines with Minimal Lithium Loss. Sep. Purif. Technol. 2021, 275, 119177 10.1016/j.seppur.2021.119177.

[ref11] Han B.; Porvali A.; Lundström M.; Louhi-Kultanen M. Lithium Recovery by Precipitation from Impure Solutions – Lithium Ion Battery Waste. Chem. Eng. Technol. 2018, 41, 1205–1210. 10.1002/ceat.201700667.

[ref12] Zheng H.; Dong T.; Sha Y.; Jiang D.; Zhang H.; Zhang S. Selective Extraction of Lithium from Spent Lithium Batteries by Functional Ionic Liquid. ACS Sustain. Chem. Eng. 2021, 9, 7022–7029. 10.1021/acssuschemeng.1c00718.

[ref13] Chen X.; Luo C.; Zhang J.; Kong J.; Zhou T. Sustainable Recovery of Metals from Spent Lithium-Ion Batteries: A Green Process. ACS Sustain. Chem. Eng. 2015, 3, 3104–3113. 10.1021/acssuschemeng.5b01000.

[ref14] Biswal B. K.; Jadhav U. U.; Madhaiyan M.; Ji L.; Yang E. H.; Cao B. Biological Leaching and Chemical Precipitation Methods for Recovery of Co and Li from Spent Lithium-Ion Batteries. ACS Sustain. Chem. Eng. 2018, 6, 12343–12352. 10.1021/acssuschemeng.8b02810.

[ref15] Kumar A.; Fukuda H.; Hatton T. A.; Lienhard J. H. Lithium Recovery from Oil and Gas Produced Water: A Need for a Growing Energy Industry. ACS Energy Lett. 2019, 4, 1471–1474. 10.1021/acsenergylett.9b00779.

[ref16] Meshram P.; Pandey B. D.; Mankhand T. R. Extraction of Lithium from Primary and Secondary Sources by Pre-Treatment, Leaching and Separation: A Comprehensive Review. Hydrometallurgy 2014, 150, 192–208. 10.1016/j.hydromet.2014.10.012.

[ref17] Toba A. L.; Nguyen R. T.; Cole C.; Neupane G.; Paranthaman M. P. U. S. Lithium Resources from Geothermal and Extraction Feasibility. Resour. Conserv. Recycl. 2021, 169, 105514 10.1016/j.resconrec.2021.105514.

[ref18] Liu J.; Zhang Y.; Miao Y.; Yang Y.; Li P. Alkaline Resins Enhancing Li+/H+Ion Exchange for Lithium Recovery from Brines Using Granular Titanium-Type Lithium Ion-Sieves. Ind. Eng. Chem. Res. 2021, 60, 16457–16468. 10.1021/acs.iecr.1c02361.

[ref19] Han B.; Anwar UI Haq R.; Louhi-Kultanen M. Lithium Carbonate Precipitation by Homogeneous and Heterogeneous Reactive Crystallization. Hydrometallurgy 2020, 195, 105386 10.1016/j.hydromet.2020.105386.

[ref20] Zhou W.; Li Z.; Xu S. Extraction of Lithium from Magnesium-Rich Solution Using Tri-n-Butyl Phosphate and Sodium Hexafluorophosphate. J Sustain Metall 2021, 7, 1368–1378. 10.1007/s40831-021-00430-7.

[ref21] Yáñez-Fernández A.; Inestrosa-Izurieta M. J.; Urzúa J. I. Concurrent Magnesium and Boron Extraction from Natural Lithium Brine and Its Optimization by Response Surface Methodology. Desalination 2021, 517, 115269 10.1016/j.desal.2021.115269.

[ref22] Matsumoto M.; Morita Y.; Yoshinaga M.; Hirose S. I.; Onoe K. Reactive Crystallization of Lithium Carbonate Nanoparticles by Microwave Irradiation of Aqueous Solution Containing CO2 Microbubbles. J. Chem. Eng. Jpn. 2009, 42, S242–S248. 10.1252/jcej.08we173.

[ref23] Sun Y.; Song X.; Wang J.; Yu J. Preparation of Li 2CO 3 by Gas-Liquid Reactive Crystallization of LiOH and CO 2. Cryst. Res. Technol. 2012, 47, 437–442. 10.1002/crat.201100571.

[ref24] Sun Y. Z.; Song X. F.; Jin M. M.; Jin W.; Yu J. G. Gas-Liquid Reactive Crystallization of Lithium Carbonate by a Falling Film Column. Ind. Eng. Chem. Res. 2013, 52, 17598–17606. 10.1021/ie402698v.

[ref25] Tian M.; Wang Z.; Cao J.; Guo J.; Gong X. Insight into Lithium Carbonate Crystallization in the Mild Reaction System LiCl-NH3·H2O-CO2 by Stabilizing the Solution with NH3·H2O. J. Cryst. Growth 2019, 520, 46–55. 10.1016/j.jcrysgro.2019.05.020.

[ref26] Zhou Z.; Liang F.; Qin W.; Fei W. Coupled Reaction and Solvent Extraction Process to Form Li2CO3: Mechanism and Product Characterization. AIChE J. 2014, 60, 282–288. 10.1002/aic.14243.

[ref27] Zhao C.; Zhang Y.; Cao H.; Zheng X.; Van Gerven T.; Hu Y.; Sun Z. Lithium Carbonate Recovery from Lithium-Containing Solution by Ultrasound Assisted Precipitation. Ultrason. Sonochem. 2019, 52, 484–492. 10.1016/j.ultsonch.2018.12.025. 30595487

[ref28] Zhu S. G.; He W. Z.; Li G. M.; Zhou X.; Zhang X. J.; Huang J. W. Recovery of Co and Li from Spent Lithium-Ion Batteries by Combination Method of Acid Leaching and Chemical Precipitation. Trans. Nonferrous Met. Soc. China 2012, 22, 2274–2281. 10.1016/s1003-6326(11)61460-x.

[ref29] Sun Y.; Song X.; Wang J.; Luo Y.; Yu J. Unseeded Supersolubility of Lithium Carbonate: Experimental Measurement and Simulation with Mathematical Models. J. Cryst. Growth 2009, 311, 4714–4719. 10.1016/j.jcrysgro.2009.09.013.

[ref30] Wang H.; Du B.; Wang M. Study of the Solubility, Supersolubility and Metastable Zone Width of Li2CO3 in the LiCl-NaCl-KCl-Na2SO4 System from 293.15 to 353.15K. J. Chem. Eng. Data 2018, 63, 1429–1434. 10.1021/acs.jced.7b01012.

[ref31] Ma Y.; Zhang Z.; Li K.; Pang D. Effects of K+, Na+, Mg2 and B4O72- Coexistence Impurities on Crystalline Characteristics of Lithium Carbonate. IOP Conf. Ser. Mater. Sci. Eng. 2019, 612, 022011 10.1088/1757-899x/612/2/022011.

[ref32] King H. E.; Salisbury A.; Huijsmans J.; Dzade N. Y.; Plümper O. Influence of Inorganic Solution Components on Lithium Carbonate Crystal Growth. Cryst. Growth Des. 2019, 19, 6994–7006. 10.1021/acs.cgd.9b00782. PMC6900547 31832024

[ref33] An J. W.; Kang D. J.; Tran K. T.; Kim M. J.; Lim T.; Tran T. Recovery of Lithium from Uyuni Salar Brine. Hydrometallurgy 2012, 117–118, 64–70. 10.1016/j.hydromet.2012.02.008.

[ref34] Jiang C.; Wang Y.; Wang Q.; Feng H.; Xu T. Production of Lithium Hydroxide from Lake Brines through Electro-Electrodialysis with Bipolar Membranes (EEDBM). Ind. Eng. Chem. Res. 2014, 53, 6103–6112. 10.1021/ie404334s.

[ref35] Um N.; Hirato T. Precipitation Behavior of Ca(OH)2, Mg(OH)2, and Mn(OH)2 from CaCl2, MgCl2, and MnCl2 in NaOH-H2O Solutions and Study of Lithium Recovery from Seawater via Two-Stage Precipitation Process. Hydrometallurgy 2014, 146, 142–148. 10.1016/j.hydromet.2014.04.006.

[ref36] Xu Z.; Zhang H.; Wang R.; Gui W.; Liu G.; Yang Y. Systemic and Direct Production of Battery-Grade Lithium Carbonate from a Saline Lake. Ind. Eng. Chem. Res. 2014, 53, 16502–16507. 10.1021/ie502749n.

[ref37] Quintero C.; Dahlkamp J. M.; Fierro F.; Thennis T.; Zhang Y.; Videla Á.; Rojas R. Development of a Co-Precipitation Process for the Preparation of Magnesium Hydroxide Containing Lithium Carbonate from Li-Enriched Brines. Hydrometallurgy 2020, 198, 105515 10.1016/j.hydromet.2020.105515.

