# Supplementary material for: Recovery of Lithium Carbonate from Dilute Li-Rich Brine via Homogenous and Heterogeneous Precipitation
Source: Ind Eng Chem Res. 2022 Aug 30;61(36):13589–602. doi: 10.1021/acs.iecr.2c01397 (PMC9480836; doi:10.1021/acs.iecr.2c01397)
Supplement: Supplementary file 1 — ie2c01397_si_001.pdf [file ie2c01397_si_001.pdf]

Supporting Information

Recovery of Lithium Carbonate from Dilute Li-Rich Brine via Homogenous and Heterogeneous Precipitation

Giuseppe Battaglia[†], *Leon Berkemeyer*[‡], *Andrea Cipollina*[†,*], *José Luis Cortina*[§], *Marc Fernandez de Labastida*[§], *Julio Lopez Rodriguez*[§], *Daniel Winter*[‡,**]

[†]Dipartimento di Ingegneria, Università degli Studi di Palermo (UNIPA), viale delle Scienze Ed.6, Palermo, 90128, Italy.

[‡]Fraunhofer Institute for Solar Energy Systems ISE, Heidenhofstraße 2, Freiburg, 79110, Germany.

[§]Chemical Engineering Department, Escola d'Enginyeria de Barcelona Est (EEBE), Universitat Politècnica de Catalunya (UPC)-BarcelonaTECH, C/Eduard Maristany 10–14, Campus Diagonal-Besòs, Barcelona, 08930, Spain.

*Email: andrea.cipollina@unipa.it

**Email: daniel.winter@ise.fraunhofer.de

S.1 Fundamentals of $Li_2CO_3(s)$ precipitation routes by Na_2CO_3 or $NaOH/CO_2$ addition

In the present work, the reactive crystallisation of $Li_2CO_3(s)$ from relatively Li-concentrated solutions was analysed studying two different separation routes: (i) addition of Na_2CO_3 solution and (ii) addition of NaOH solution and CO_2 insufflation.

In the first case, Li^+ and CO_3^{2-} ions react yielding the poorly soluble $Li_2CO_3(s)$, according to Eq. (S1).

$$2Li^+(aq) + CO_3^{2-}(aq) \rightarrow Li_2CO_3(s) \tag{S1}$$

Li_2CO_3 precipitation using Na_2CO_3 has been widely investigated in the literate and various parameters affecting Li_2CO_3 recovery were studied: the reaction temperature, the solution pH, the initial Li^+ concentration, the presence of dissolved salts, the Na_2CO_3 addition flow rate and the stirring speed[1].

On the other hand, the NaOH solution and CO_2 insufflation precipitation route has been less studied in the literature. In the process, first, CO_2 has to dissolve in the Li^+ solution, Eq. (S2). Then, the dissolved $CO_2(aq)$ reacts with OH^- ions to form bicarbonates (HCO_3^{-1}), Eq. (S3). Bicarbonates further react with OH^- ions leading to carbonates, Eq. (S4). Finally carbonates and lithium ions precipitate as Li_2CO_3, Eq. (S5). A continuous CO_2 insufflation, however, can decrease solution pH affecting the CO_2/HCO_3 equilibrium causing the re-dissolution of the precipitated Li_2CO_3 in the form of the soluble $LiHCO_3$, Eq. (S6).

$$CO_2(g) \rightarrow CO_2(aq) \tag{S2}$$

$$CO_2(aq) + OH^-(aq) \rightarrow HCO_3^-(aq) \tag{S3}$$

$$HCO_3^-(aq) + OH^-(aq) \rightarrow H_2O(l) + CO_3^{2-}(aq) \quad \text{(S4)}$$

$$2\,Li^+(aq) + CO_3^{2-}(aq) \rightarrow Li_2CO_3(s) \quad \text{(S5)}$$

$$CO_2(aq) + Li_2CO_3(s) + H_2O(l) \rightarrow 2HCO_3^-(aq) + 2Li^+(aq) \quad \text{(S6)}$$

The complex set of simultaneous reactions, taking place during the Li_2CO_3(s) precipitation process in a gas-liquid reaction system, makes the process more difficult to be analysed and controlled with respect to the classical use of Na_2CO_3 separation route[2]. In the precipitation gas-liquid system, several parameters have to be carefully controlled, e.g. the reaction temperature, the solution pH, the CO_2(g) volume flow rate and the bubble size [3]. The reaction temperature does not only influence the solubility of Li_2CO_3, but also the CO_2(g) dissolution velocity. Increasing temperature decreases Li_2CO_3 solubility that, in turn, induces a faster CO_2(g) consumption pushing the CO_2(g) dissolution due to the lower CO_2(g) bulk concentration in the solution. On the other hand, it has also to note that CO_2(g) solubility decreases. A critical parameter is the solution pH. As shown from Eq. (S6), the final pH affects the re-carbonation equilibrium of Li_2CO_3, thus the time point when CO_2 is stopped must be properly controlled to avoid excessive re-carbonation of Li_2CO_3, which causes excessive product loss. Bubbles' size is also an important parameters: small bubbles enhances CO_2(g) mass transport, thanks to large specific surface area, accelerating the process[4].

S.1.1 Li_2CO_3(s) solubility in pure water system

In comparison to most salts, the solubility of Li_2CO_3(s) decreases increasing temperature. The solubility constant (K_{sp}) at 20 ºC is $5.3\cdot10^{-4}$ and $8.9\cdot10^{-5}$ at 90 ºC according to the HSC data of Metso-Outotec. In the present work, an initial Li^+ concentration of about 4000 ppm was employed in all experiments in accordance with expectation of the SEArcularMINE project chain. At such initial Li^+ concentration, the maximum theoretical Li^+ recovery can vary from

59 % at 50 °C to 69 % at 80 °C [5], when using reactants stoichiometric amounts. It should be noted that, at an initial Li^+ concentration of 4000 ppm, only Li_2CO_3 could be studied as a viable route for Li^+ recovery due to the high solubility of LiOH(s) in water, i.e. ~127,000 ppm at 20 °C [6] with respect to that of Li_2CO_3 (~13,000 ppm at 20 °C).

S.1.2 Influence of Inorganic Solution Components on $Li_2CO_3(s)$ solubility

The presence of dissolved ions in Li^+ solutions has been found to influence Li_2CO_3 solubility. Sun et al.[1] reported a Li_2CO_3 solubility decrease after the dissolution of NaCl and KCl salts (salt-out effects), while Li_2CO_3 solubility increased dissolving Na_2SO_4 salts (salt-in effect). Cheng et al [5] studied the $Li_2CO_3(s)$ solubility in Na^+, K^+, Li^+, Cl^- brines in a temperature range between 20 and 90 °C by the isothermal dissolution method. Li_2CO_3 was found to be more soluble in NaCl and KCl single added salt systems and even more soluble in mixtures of dissolved NaCl and KCl solutions. Wang et al. [7] measured the solubility, the super solubility, and the metastable zone width of Li_2CO_3 in mixed LiCl−NaCl−KCl−Na_2SO_4 solutions in a temperature range from 20 to 80 °C employing the isothermal dissolution method and the turbidity technology. Results were in accordance with those reported by Sun et al. [1]. Ma et al. [8] studied the crystallization of lithium carbonate in LiCl solutions containing other salts mimicking Chine's Salt Lakes ones. It was observed that lithium carbonate solubility decreased increasing composition of K^+, Na^+, Mg^{2+} and $B_4O_7^{2-}$ ions. At the same time, as salts concentration increased, $Li_2CO_3(s)$ particle sizes and purity decreased. King et al.[9] investigated the $Li_2CO_3(s)$ precipitation process also in the presence of divalent cations, i.e. Mg^{2+} and Ca^{2+}, in LiBr solutions. Even at low Mg^{2+} concentration, Li^+ precipitation was found to be considerably hindered. Furthermore, the presence of divalent ions reduced $Li_2CO_3(s)$ crystals growth and purity.

S.2 Supporting tables

Tables listing additional information about the experimental tests discussed in the manuscript.

Table S1 Chemical reagents characteristics employed for Li+ precipitation analysis.

Compound	Formula	Manufacturer	Purity
Lithium chloride	$LiCl$	Carl Roth	≥99 %
Sodium carbonate	Na_2CO_3	VWR	99.7 %
Strontium chloride hexahydrate	$SrCl_2*6H_2O$	Carl Roth	≥99 %
Potassium chloride	KCl	Carl Roth	≥99 %
Sodium chloride	$NaCl$	Merck	≥99 %
Nitric acid	HNO_3	Carl Roth	69 %
Rubidium chloride	$RbCl$	Carl Roth	≥99 %
Sodium hydroxide	$NaOH$	Carl Roth	≥99 %
Magnesium chloride	$MgCl_2$	Carl Roth	≥98.5 %
Calcium chloride	$CaCl_2$	Carl Roth	≥94 %
Ethanol	CH_3CH_2OH	Carl Roth	≥70 %
Carbon dioxide	CO_2	Linde	99.9 %

Table S2 Reacting quantities for Li_2CO_3 precipitation tests at different $[CO_3^{2-}]/[Li^+]$.

CO_3^{2-}/ Li$^+$ Ratio	Li$^+$ concentration in the feed solution [ppm]	LiCl feed solution volume [mL]	2.0 M Na_2CO_3 volume [mL]	Li$^+$ concentration in the total volume [ppm]
0.25	4330	115	9	4010
0.5	4780	105	18	4080
0.75	5280	95	28	4080
1	5820	86	36	4100
2	10100	50	72	4140

Table S3 Calculated ionic strength for 0.70 M LiCl solutions, before Na_2CO_3 solution addition, with and without NaCl and KCl salts.

Investigated solutions	Ionic strength *I* [M]
0.70 M LiCl	0.70
0.70 M LiCl and 1.5 M KCl	2.20
0.70 M LiCl and 2.0 M NaCl	2.70
0.70 M LiCl and 1.5 M KCl + 2.0 M NaCl	4.20

Table S4 Divalent cations concentration in LiCl solutions with 2.0 M NaCl and 1.5 M KCl (before addition of Na_2CO_3 solution). In each solution, Li^+ concentration after carbonate dosing was ~4000 ppm.

Investigated solutions before Na_2CO_3 addition	Ionic strength *I* [mol/L]
0.70 M LiCl, 1.5 M KCl, 2.0 M NaCl	4.20
0.70 M LiCl, 1.5 M KCl, 2.0 M NaCl + 0.17 M $CaCl_2$	4.70
0.70 M LiCl, 1.5 M KCl, 2.0 M NaCl + 0.25 M $MgCl_2$	4.95
0.70 M LiCl, 1.5 M KCl, 2.0 M NaCl + 0.17 M $SrCl_2$	4.70
0.70 M LiCl, 1.5 M KCl, 2.0 M NaCl + 0.17 M $CaCl_2$ + 0.25 M $MgCl_2$ + 0.17 M $SrCl_2$	5.95

Table S5 Reacting solutions at different OH^-/Li^+ ratios.

OH^-/Li^+ Ratio	LiCl volume [mL]	NaOH volume [mL]	Li^+ concentration after NaOH addition [ppm]
1	110	9.0	4290
1.5	110	13	4180
2	110	18	3900
4	110	47	4165

References

(1) Sun, Y.; Song, X.; Wang, J.; Luo, Y.; Yu, J. Unseeded Supersolubility of Lithium Carbonate: Experimental Measurement and Simulation with Mathematical Models. *J. Cryst. Growth* **2009**, *311* (23–24), 4714–4719. https://doi.org/10.1016/j.jcrysgro.2009.09.013.

(2) Tian, M.; Wang, Z.; Cao, J.; Guo, J.; Gong, X. Insight into Lithium Carbonate Crystallization in the Mild Reaction System LiCl-NH3·H2O-CO2 by Stabilizing the Solution with NH3·H2O. *J. Cryst. Growth* **2019**, *520*, 46–55. https://doi.org/10.1016/j.jcrysgro.2019.05.020.

(3) Sun, Y.; Song, X.; Wang, J.; Yu, J. Preparation of Li 2CO 3 by Gas-Liquid Reactive Crystallization of LiOH and CO 2. *Cryst. Res. Technol.* **2012**, *47* (4), 437–442. https://doi.org/10.1002/crat.201100571.

(4) Han, B.; Anwar UI Haq, R.; Louhi-Kultanen, M. Lithium Carbonate Precipitation by Homogeneous and Heterogeneous Reactive Crystallization. *Hydrometallurgy* **2020**, *195*, 105386. https://doi.org/10.1016/j.hydromet.2020.105386.

(5) Cheng, W.; Li, Z.; Cheng, F. Solubility of Li2CO3 in Na-K-Li-Cl Brines from 20 to 90 °c. *J. Chem. Thermodyn.* **2013**, *67*, 74–82. https://doi.org/10.1016/j.jct.2013.07.024.

(6) Stephan, E. F.; Miller, P. D. Solubility of Lithium Hydroxide in Water and Vapor Pressure of Solutions above 220° F. *J. Chem. Eng. Data* **1962**, *7* (4), 501–505. https://doi.org/10.1021/je60015a018.

(7) Wang, H.; Du, B.; Wang, M. Study of the Solubility, Supersolubility and Metastable Zone Width of Li2CO3 in the LiCl-NaCl-KCl-Na2SO4 System from 293.15 to 353.15K. *J. Chem. Eng. Data* **2018**, *63* (5), 1429–1434. https://doi.org/10.1021/acs.jced.7b01012.

(8) Ma, Y.; Zhang, Z.; Li, K.; Pang, D. Effects of K+, Na+, Mg2 and B4O72- Coexistence Impurities on Crystalline Characteristics of Lithium Carbonate. *IOP Conference Series: Materials Science and Engineering* **2019**, *612*, 022011. https://doi.org/10.1088/1757-899X/612/2/022011.

(9) King, H. E.; Salisbury, A.; Huijsmans, J.; Dzade, N. Y.; Plümper, O. Influence of Inorganic Solution Components on Lithium Carbonate Crystal Growth. *Cryst. Growth Des.* **2019**, *19* (12), 6994–7006. https://doi.org/10.1021/acs.cgd.9b00782.
